# Self‐Assembly of Atomically Precise Silver Nanoclusters in Crowded Colloids into Ultra‐Long Ribbons with Tunable Supramolecular Chirality

**DOI:** 10.1002/advs.202305102

**Published:** 2023-11-20

**Authors:** Wenjuan Wang, Tong Liu, Ting Zhao, Di Sun, Hongguang Li, Pengyao Xing, Xia Xin

**Affiliations:** ^1^ Key Laboratory of Colloid and Interface Chemistry Ministry of Education National Engineering Research Center for Colloidal Materials School of Chemistry and Chemical Engineering Shandong University Ji'nan 250100 P. R. China

**Keywords:** colloids, metal nanoclusters, ribbons, self‐assembly, supramolecular chirality, η

## Abstract

Atomically precise metal nanoclusters (NCs) emerge as fascinating synthons in self‐assembled materials. The self‐assembly of metal NCs are highly sensitive to the environment because they have an inorganic‐organic hybridized structure and a relatively complicated conformation. Here, it is shown that when confined in crowded colloids, a water‐soluble Ag_9_‐cored nanocluster (Ag_9_‐NC) can self‐ assemble into ultra‐long (up to millimeters) and photoluminescent ribbons with high flexibility. The ribbon contains rectangularly organized columns of Ag_9_‐NCs and can undergo secondary self‐assembly to form bundled and branched structures. Formation of ribbons is observed in all the tested colloids, including lyotropic liquid crystals and disordered, three‐dimensional network. The high viscosity/elasticity of the crowded colloids weakens gravity‐induced sedimentation of the ribbons, leading to the formation of an interesting class of inorganic‐organic composite materials where the hard Ag‐containing skeleton strengthens the soft matter. The simultaneously occurring symmetry breaking during the self‐assembly of Ag_9_‐NCs gives uncontrolled supramolecular chirality, which can be tuned through the majority rule and soldier‐and‐sergeant rule by the introduction of chiral seeds. The regulated chirality and the intrinsic photoluminescence of the Ag_9_‐NCs ribbons impart the composite material circularly polarized luminescence, opening the door for a variety of potential applications.

## Graphical abstract

A negatively‐charged silver nanocluster (Ag_9_‐NC) can self‐assemble into ultralong ribbons with high flexibility in nonionic as well as anionic‐crowded colloids. The uncontrolled supramolecular chirality originated from spontaneous symmetry breaking and can be made homochiral with the addition of chiral seeds. Combined with the aggregation‐induced emission of Ag_9_‐NCs, a new class of inorganic–organic composite materials with circularly polarized luminescence and improved mechanical strength are finally obtained.

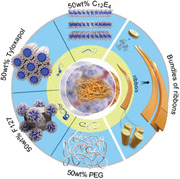

## 1 Introduction

Atomically precise metal nanoclusters (NCs) are constituted by a few to hundreds of metal atoms with a ligand shell and sizes ranging from one to several nanometers.^[^ ^1^, ^2^, ^3^ ^]^ Depending on their devisable and diversified structures, metal NCs possess interesting electronic properties, with modulated and finely tailored absorption, emission, and magnetic properties, which have led to a wide scope of applications in catalysis, display, biomedicine, energy, and environmental protection.^[^ ^4^, ^5^, ^6^, ^7^, ^8^, ^9^ ^]^ Being a class of nanometer‐sized building blocks, metal NCs also exhibit great potential in nanoscience and supramolecular chemistry. The inorganic‐organic hybridized nature of metal NCs makes them different from small organic molecules, polymers, and biomacromolecules. The rather complicated structures of metal NCs, normally appearing in a 3D form, provide plenty of room for thermodynamic and kinetic control of the self‐assembly. Compared to nanoparticles without atomic precision, metal NCs are strictly monodispersed with specific atoms and packing, which provides a good opportunity for deep studying the structure‐property relationship of the self‐assemblies. To date, various metal NCs have been synthesized, which were covered by diversified ligands. For metal NCs with precise structures confirmed by single crystal analysis, the ligands used are normally small molecules. As the active part (typically polar groups) will be consumed by coordination with metal atoms/ions, the so‐obtained metal NCs are typically hydrophobic. Pioneering work on these metal NCs has led to encouraging discoveries with the production of abundant nanostructures and soft materials.^[^ ^10^, ^11^, ^12^, ^13^, ^14^ ^]^

**Scheme 1:**
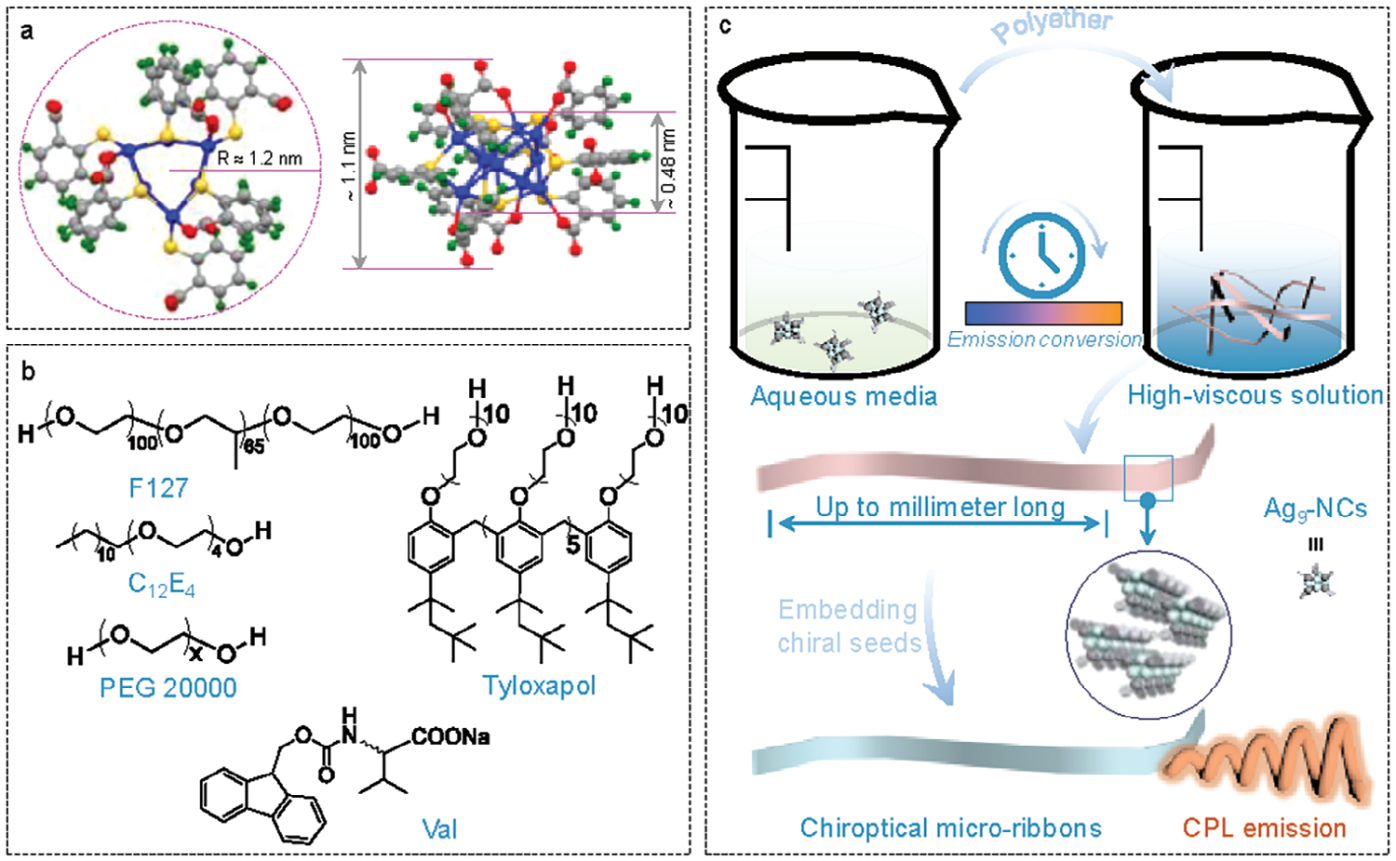
a) Top (left) and side (right) view of Ag_9_‐NC, obtained from single crystal analysis. Values of typical distances are given. The dashed circle in a is a guide for the eyes. Blue: Ag, yellow: S, red: O, grey: C. green: H. b) Structures of the polyethers and chiral seeds adopted in the current study. c) Schematic representation of the self‐assembly of Ag_9_‐NCs in crowded colloids and the subsequent chiral regulation by seeds.

To enrich the noncovalent interaction among the clusters and increase the sensitivity of the metal NCs toward the environment during self‐assembly, it would be necessary to impart the metal NCs surplus functional groups. For this purpose, the selection of polydentate ligands is necessary. For example, we have demonstrated that silver NC containing six silver atoms (Ag_6_‐NC) could be obtained by using mercaptonicotinic acid as a ligand, which has six peripheral carboxylic groups surrounding the cluster.^[^ ^15^ ^]^ Driven by the rich inter‐cluster interactions including the electrostatic interaction and hydrogen bonding, Ag_6_‐NC exhibits interesting self‐assembly behavior in aqueous and protonic solutions.^[^ ^16^, ^17^, ^18^ ^]^ Another example is silver NC containing nine silver atoms (Ag_9_‐NC, **Scheme** **1**a) with thiosalicylic acid as ligands, which have three peripheral carboxy groups. Compared to Ag_6_‐NC, Ag_9_‐NC has a lower water solubility due to the decreased number of the peripheral carboxylic groups while increased number of the silver atoms in the core. Thus, it can be dissolved only in an alkaline solution, which makes it highly sensitive to the perturbation of the environment. Upon the addition of acids, crystallization‐induced self‐assembly (CISA)^[^ ^19^, ^20^ ^]^ was triggered due to the fast change of the pH, leading to the formation of crystallized self‐assemblies.^[^ ^21^, ^22^, ^23^, ^24^ ^]^ CISA could also be induced by the modification of the solvent by the introduction of a protonic solvent (ethanol, EtOH),^[^ ^25^ ^]^ or by coordination of the peripheral carboxylic groups with secondary metal ions (such as Ba^2+^).^[^ ^26^ ^]^

These preliminary results proved that Ag_9_‐NC is a powerful building block in self‐assembly. Structural analysis of the self‐assemblies revealed that the internal organization is highly dependent on the environment and the aggregation pathway of Ag_9_‐NCs. In a solvent‐free state, the crystallized self‐assemblies have quite a similar organization to that of the lyophilized stock solution of Ag_9_‐NCs.^[^ ^24^ ^]^ In the gels induced by EtOH, the self‐assemblies exhibit a high degree of crystallization but a poor phase purity.^[^ ^25^ ^]^ In Ba^2+^‐based gel, a well‐defined chiral cubic (I*) phase was observed with a space group of I4132 (Q214), but the degree of order is not high seen from the relatively large peak width at half‐height.^[^ ^26^ ^]^

To get a deep understanding of the self‐assembly of metal NCs and to further expand the applications of the self‐assemblies, in this work, we created a new environment to tune the self‐assembly of Ag_9_‐NCs. When confined in highly concentrated aqueous solutions of polyether‐based surfactants (C_12_E_4_, Tyloxapol, and F127, Scheme 1b) or macromolecule (PEG, Scheme 1b), the hydrophilic part of Ag_9_‐NC was dehydrated and the local concentration of Ag_9_‐NC was increased, which successfully triggered CISA. The highly viscous colloids not only provided an ideal microenvironment for the elongation of the self‐assemblies but also weakened the gravity‐induced sedimentation, leading to the formation of ultralong (up to millimeters) and photoluminescent (PL) ribbons with high flexibility. Importantly, the rather slow kinetics aided by the highly viscous microenvironment give enough time for the self‐assembly of Ag_9_‐NCs, leading to the formation of a columnar rectangular (Col_r_) phase. By applying the majority rule and soldier‐and‐sergeant rule, the spontaneously‐occurred supramolecular chirality during the self‐assembly, which is irregular and hard to control, was made homochiral by the introduction of chiral seeds (Fmoc‐Val‐ONa and Fmoc‐Ala‐ONa, abbreviated to Val and Ala, respectively, Scheme 1b). Combining the intrinsic photoluminescence of the self‐assembled Ag_9_‐NCs, inorganic–organic hybrid materials with circularly polarized luminescence (CPL) were finally obtained (see Scheme 1c for the illustration of the whole self‐assembly process).

## 2 Results and Discussion

**Figure 1:**
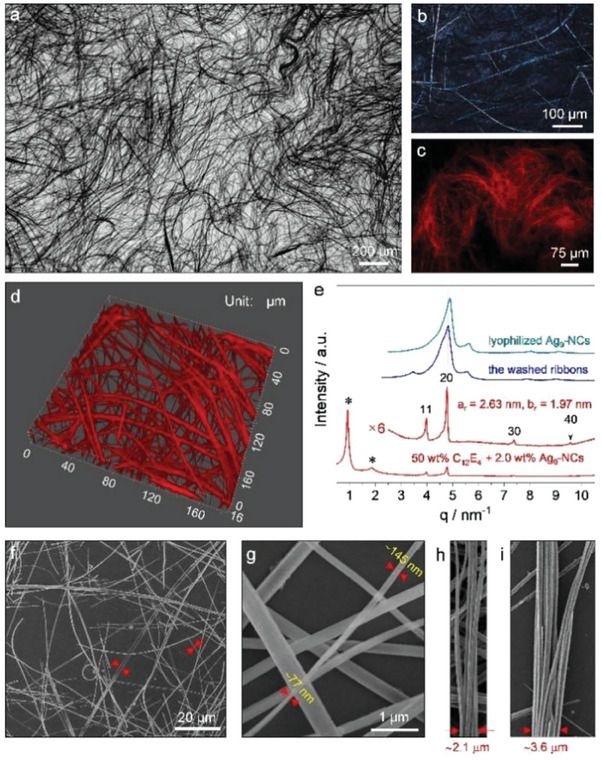
Morphologies and structures of the self‐assembled Ag_9_‐NCs (2.0 wt.%) in the lamellar LLC phase of C_12_E_4_ (50 wt.%). a) Optical microscopy, b) POM, and c) fluorescent images of the self‐assemblies embedded inside the LLC phase. d) 3D CLSM image of the self‐assemblies embedded in the LLC phase. The voids will be occupied by surfactant and water molecules, which are invisible under the microscope. e) SAXS patterns of the self‐assemblies before and after washing. Results from lyophilized Ag_9_‐NCs are also given for comparison. The dashed line is a guide for the eyes. f–i) SEM images of the self‐assemblies after washing.

We first investigated the self‐assembly of Ag_9_‐NCs in aqueous solution of C_12_E_4_ with a concentration of 50 wt.%. At this concentration, C_12_E_4_ itself forms a lyotropic liquid crystal (LLC) phase with lamellar organization proved by polarized optical microscopy (POM) observations and small‐angle X‐ray scattering (SAXS) measurements (Figure S1, Supporting Information). After Ag_9_‐NCs were introduced and the sample was aged for a sufficiently long time (over one week), elongated nanostructures formed which were evenly distributed inside the LLC phase. As seen from the optical microscopy image in **Figure** **1**a, the nanostructures occupy the whole area of the image with a length of up to millimeters. Entanglement and bending were noticed, indicating their high flexibility. Optical anisotropy was noticed when crossed polarizers were applied, especially for the thick nanostructures (Figure 1b). Ag_9_‐NCs exhibit aggregation‐induced emission (AIE) with intrinsic red emission, which makes the nanostructures visible under a fluorescence microscope (Figure 1c). More structural details of the nanostructures were obtained from 3D reconstructed confocal laser scan microscopy (CLSM) images. As presented in Figure 1d and the supporting movie, the diameters of the nanostructures are polydisperse. They have a solid structure with tabular cross‐sections, indicating that they are actually ribbons. Among them, furcation of a thick ribbon into smaller ones and coalesce of thin ribbons into a big one occurred quite often. This feature, combining the bending of individual ribbons, led to the formation of a 3D network. The internal structure of the ribbons was probed by SAXS measurements (Figure 1e). Four peaks were observed in addition to those of the lamellar phase (marked by the stars), which can be assigned to the (11), (20), (30), and (40) planes of a columnar rectangular (Col_r_) phase^[^ ^27^ ^]^ with a lattice parameter of *a*_r_ = 2.63 nm and *b*_r_ = 1.97 nm.

In our previous work, we have shown that Ba^2+^‐induced self‐assemblies of Ag_9_‐NCs underwent a structural transition during the post‐treatment by washing and drying.^[^ ^26^ ^]^ In the current study, the same phenomenon was noticed. As seen in Figure 1e, the SAXS curve of the ribbons lost the feature of the Col_r_ phase once they were washed out of the LLC phase followed by drying. The curve became quite similar to that of the lyophilized Ag_9_‐NCs with only a slight peak shift toward small *q* values (< 0.1 nm^−1^), indicating that Ag_9_‐NCs are the main components of the dried ribbons. This conclusion gained further support from the rather similar curves of the washed ribbons and lyophilized Ag_9_‐NCs from X‐ray photoelectron spectroscopy (XPS) measurements (Figure S2, Supporting Information), as well as energy dispersive spectroscopy (EDS) analysis on the ribbons where elements of Ag and S were detected besides C and O (Figure S3, Supporting Information). However, from Fourier transform infrared (FTIR) measurements, we realized that the spectrum of the washed ribbons also contained signals from C_12_E_4_ (Figure S4, Supporting Information), indicating that C_12_E_4_ also attended in the self‐assembly of Ag_9_‐NCs, which could not be fully removed by washing. In addition, the peaks ≈1457, 1282, and 1236 cm^−1^, which originated from C_12_E_4_, and those in between 768–616 cm^−1^, which came from lyophilized Ag_9_‐NCs, shift to lower wavenumbers. These observations indicate that noncovalent interaction exists between C_12_E_4_ and Ag_9_‐NCs, which led to better thermal stability of the washed ribbons compared to that of the lyophilized Ag_9_‐NCs confirmed by thermogravimetric analysis (TGA, Figure S5, Supporting Information).

Despite the internal structural transition induced by washing and drying, the ribbons basically retained their morphologies. From the scanning electron microscopy (SEM) image shown in Figure 1f, ribbons were clearly observed. A typical one with a width of ≈1.31 µm has been marked between the arrowheads. Also marked is a thin one with a width of only ≈0.17 µm (between the arrows). SEM observation with a higher magnification shows the twisting of a small ribbon, with the widest and narrowest places to be ≈145 and ≈77 nm, respectively (Figure 1g). Examination of thick ribbons showed that they are formed by the secondary self‐assembly of smaller ones. Two typical images exhibiting this situation are given in Figure 1h,i. The structural features of the ribbons were also proved by atomic force microscopy (AFM) and transition electron microscopy (TEM) observations (Figures S6 and S7, Supporting Information).

**Figure 2:**
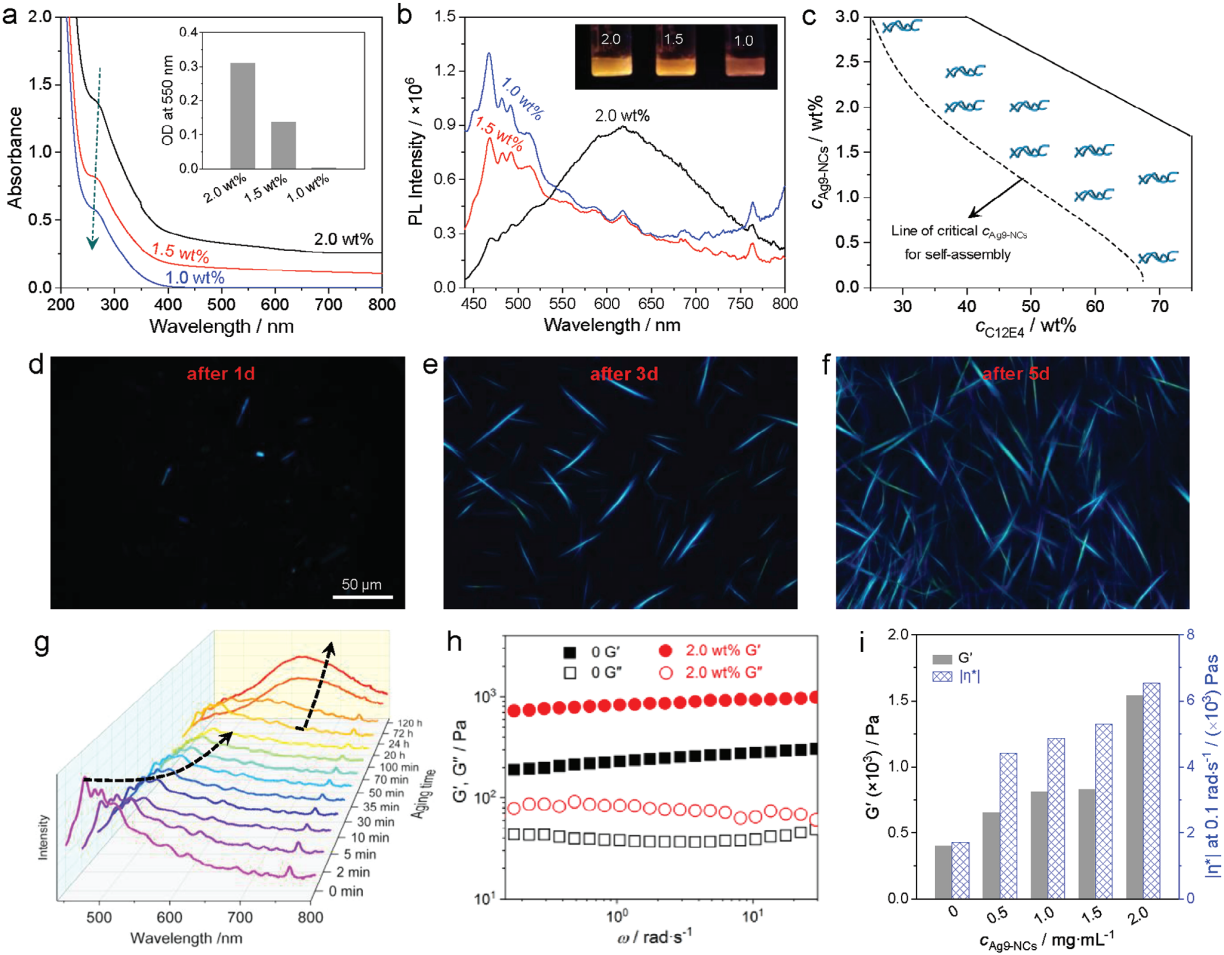
UV–vis absorption a) and emission at an optimized excitation wavelength of 420 nm b) of the lamellar LLC phase of C_12_E_4_ (50 wt.%) containing varying amounts of Ag_9_‐NCs as indicated. Inset in a are the statistics of the optical density (O.D.) at 550 nm and insets in b are photos of the three selected samples under 365 nm UV irradiation. c) A sample matrix for C_12_E_4_/Ag_9_‐NCs/H_2_O ternary system. The intertwined symbols denote the nanoribbons. d–f) POM images recorded at different times after sample preparation for Ag_9_‐NCs (2.0 wt.%) in C_12_E_4_ (50 wt.%). The images were recorded with the same magnification (the scale is given in image d). g) Time‐dependent emission of Ag_9_‐NCs (2.0 wt.%) in C_12_E_4_ (50 wt.%). The two arrows are guides for the eyes. h) Variations of the elastic modulus G’ and viscous modulus *G*” as a function of the angular frequency ω for Ag_9_‐NCs (2.0 wt.%) in C_12_E_4_ (50 wt.%). For comparison, data from the sample without Ag_9_‐NCs are also given. i) Statistics of G’ and complex viscosity |h*| recorded at 0.1 rad•s^−1^ for the C_12_E_4_ (50 wt.%) containing varying amount of Ag_9_‐NCs.

Formation of the ribbons is influenced by the concentrations of both Ag_9_‐NCs (*c*_Ag9‐NCs_) and C_12_E_4_ (*c*_C12E4_). At a fixed concentration of *c*_C12E4_ of 50 wt.%, lowering *c*_Ag9‐NCs_ from 2.0 wt.% to 1.5 wt.% caused an obvious decrease in the number density of the ribbons proved by polarized optical microscopy (POM) observations (Figure S8, Supporting Information). From SAXS patterns, peaks from the Col_r_ phase could hardly be detected (Figure S9, Supporting Information). The less pronounced self‐assembly of Ag_9_‐NCs at lower concentrations caused obvious changes in the optical properties of the composite materials. As seen in **Figure** **2**a, the sample of 2.0 wt.% Ag_9_‐NCs/50 wt.% C_12_E_4_ showed absorbance across the UV and visible regions with a shoulder peak ≈265 nm. When *c*_Ag9‐NCs_ decreased to 1.5 wt.%, a hypochromatic shift of the peak (by ≈4 nm) was observed together with an obvious decrease in absorption. When *c*_Ag9‐NCs_ further decreased to 1.0 wt.%, the peak underwent a further hypochromatic shift (by ≈1 nm) and the sample became optically transparent above 400 nm. From PL measurements, the sample of 2.0 wt.% Ag_9_‐NCs/50 wt.% C_12_E_4_ exhibited an optimized excitation at ≈420 nm with a broad emission covering the yellow and red regions (Figure 2b, Figure S10, Supporting Information). When *c*_Ag9‐NCs_ decreased, the intensity in the long wavelengths weakened and the emission transferred to the blue region, which was assigned to the emission from the peripheral ligands.^[^ ^26^ ^]^ The influence of *c*_C12E4_ on the self‐assembly of Ag_9_‐NCs is also obvious. At a fixed *c*_Ag9‐NCs_ of 2.0 wt.%, ribbons could not be observed by POM if *c*_C12E4_ was below 40 wt.%. Examination of other compositions showed that the self‐assembly of Ag_9_‐NCs was heavily dependent on *c*_C12E4_. A sample matrix could be obtained by POM observations, which are given in Figure 2c (also see Figure S11, Supporting Information, which contains typical images). One can see that a larger *c*_C12E4_ corresponded to a lower critical *c*_Ag9‐NCs_ for the formation of ribbons, indicating that a more crowded colloidal environment is beneficial for the self‐assembly of Ag_9_‐NCs.

The kinetics involved in the self‐assembly were tracked by time‐dependent POM observations using the sample with *c*_C12E4_ = 50 wt.% and *c*_Ag9‐NCs_ = 2.0 wt.% as an example. The image obtained 1 day after sample preparation showed only quite a limited number of anisotropic, short ribbons (Figure 2d). During aging, both the number and length of the ribbons increased (Figure 2e,f). The growth of ribbons induced a continuous change of the photoluminescence, with the emission gradually shifting from the blue region to the red region (Figure 2g). Compared to EtOH‐induced ^[^ ^25^ ^]^ and Ba^2+^‐induced gels,^[^ ^26^ ^]^ the time scales noticed in the self‐assembly of Ag_9_‐NCs in the LLC phase of C_12_E_4_ are significantly longer. Probably, the high viscosity of the system and the presence of the surfactant bilayers set barriers and retarded the self‐assembly. In this case, Ag_9_‐NCs have sufficient time to tune their conformation to better fit in the self‐assemblies, accounting for the formation of ribbons with high internal order.

The formation of hard nanostructures (ribbons) embedded in the soft matters (LLC phase) enhances the mechanical strength of the LLC phase has been reinforced by the presence of the ribbons. At *c*_Ag9‐NCs_ of 2.0 wt.%, the elastic modulus (*G*’) of the LLC phase of 50 wt.% C_12_E_4_ was improved by ≈3.6 folds (Figure 2h, Figure S12, Supporting Information), and the complex viscosity (|h*|) was increased by ≈4.3 folds (Figure S13, Supporting Information). Further examination revealed that both *G*’ and |h*| increased steadily with increasing *c*_Ag9‐NCs_ (Figure 2i).

Thus, reinforcement also occurred at low cAg9‐NCs, despite the fact that the ribbons were hard to capture by imaging studies under such circumstances.

**Figure 3:**
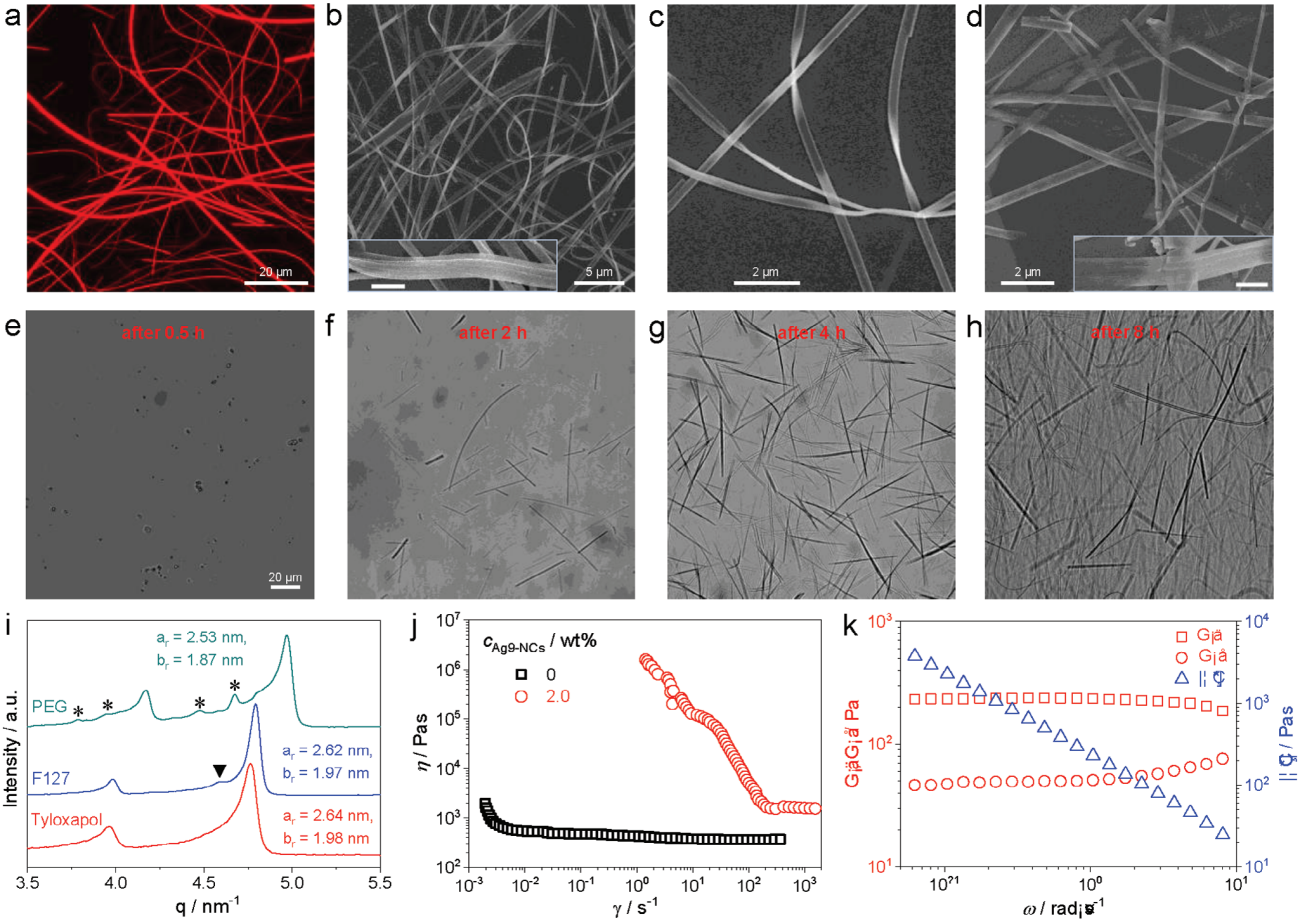
a–d) Typical results from imaging studies on the self‐assembled Ag_9_‐NCs (2.0 wt.%) in 50 wt.% of Tyloxapol (a, CLSM image), F127 (b, c, SEM images) and PEG (d. SEM image). Inset of b highlights the stacking of the nanoribbons to form a bigger one, while that of d highlights the joint of four nanoribbons to form a wider one. In both cases, the scale bar corresponds to 1 mm. e–h) Optical microscopy images recorded at different times show the evolution of the self‐assemblies of Ag_9_‐NCs (2.0 wt.%) in F127 (50 wt.%). i) Comparison of the scattering peaks in the range of 3.5–5.5 nm^−1^, which contains the (11) and (20) planes of the Col_r_ phase, for Ag_9_‐NCs (2.0 wt.%) in aqueous solutions formed by the three polyethers as indicated (50 wt.%). j) Variation of the apparent viscosity as a function of shear rate for the sample containing 2.0 wt.% Ag_9_‐NCs and 50 wt.% PEG. For comparison, data from the sample without Ag_9_‐NCs are also given. k) Variations of *G*', *G*'', and |η*| as a function of ω for the sample containing 2.0 wt.% Ag_9_‐NCs and 50 wt.% PEG.

It is well‐known that surfactants can form various mesophases in aqueous solutions with diverse structures.^[^ ^28^, ^29^, ^30^ ^]^ Next issue is whether or not the self‐assembly of Ag_9_‐NCs into ribbons is specific to the LLC phase of C_12_E_4_. To figure it out, other two LLC phases formed also by nonionic surfactants were selected, including the hexagonal phase formed by Tyloxapol (Figure S14, Supporting Information) and the cubic phase formed by Pluronic F127 (Figure S15, Supporting Information). An amorphous phase formed by PEG, which is a nonionic, fully hydrophilic polymer lacking amphiphilicity, was also selected. Comparison between the rheological properties of the four samples with a concentration of 50 wt.% revealed big differences in their mechanical strength, as seen in **Table** **1**. The hexagonal phase shows the highest elastic modulus while the cubic phase has the largest viscosity. The amorphous phase formed by PEG has the smallest viscosity and lacks elasticity. However, it exhibits a less pronounced shear‐thinning behavior with ≈24% loss of viscosity when the shear rate was increased from 0.1 to 100 s^−1^. The self‐assembly of Ag_9_‐NCs in the above three colloids was fully investigated. At a fixed concentration of the colloids of 50 wt.% and a fixed *c*_Ag9‐NCs_ of 2.0 wt.%, imaging studies showed that ribbons formed within all of them (**Figure** **3**a–d, Figure S16, Supporting Information). Morphological analysis of these structures revealed two more assembly modes of the ribbons, i.e., stacking (inset of Figure 3b) and cross‐joining (inset of Figure 3d). Further investigations on other concentrations showed that ribbon formation was easier in these three colloids compared to the system of C_12_E_4_. For example, at *c*_Tyloxapol_ = 50 wt.%, the formation of ribbons was already obvious at *c*_Ag9‐NCs_ = 1.5 wt.% (Figure S17, Supporting Information). Even at *c*_Ag9‐NCs_ = 1.0 wt.%, the presence of ribbons could be distinguished despite the low number density (Figure S18, Supporting Information). For another example, when *c*_Ag9‐NCs_ are fixed at 2.0 wt.%, a considerable amount of ribbons was observed in 30 wt.% F127 (Figure S19, Supporting Information), which is not the case for C_12_E_4_ (see Figure 2c). For PEG solutions, formation of ribbons was also obvious when *c*_PEG_ reached 40 wt.% at *c*_Ag9‐NCs_ = 2.0 wt.% (Figure S20, Supporting Information). Time‐dependent studies on F127‐based system revealed that the ribbon formation exhibited relatively fast kinetics. As shown in Figure 3e–h, large amount of ribbons appeared only 8 h after sample preparation for F127‐based system, which is much shorter than the time scale noticed in the lamellar phase of C_12_E_4_. These observations showed that although the formation of ribbons is equally successful for Ag_9_‐NCs, the parameters of each colloid could also modify the process of self‐assembly.

**Table 1:** Parameters for C_12_E_4_, Tyloxapol, F127, and PEG as well as their aqueous solutions (at a fixed concentration of 50 wt.%).

		C_12_E_4_	Tyloxapol	F127	PEG
Amphiphilic?		Yes	Yes	Yes	No
Percentage of the hydrophilic moiety		≈ 53.3%	≈ 70.3%	≈ 70.5%	100%
Type of the mesophase		Lamellar	Hexagonal	Cubic	Amorphous
|η*| / mPas	at 0.1 rad × s^−1^	1736	1.37×10^5^	1.87×10^8^	482.6^a)^
	at 100 rad × s^−1^	2.86	507.4	1.89 ×10^5^	365.8^b)^
G' at 1.0 Hz / Pa		708.7	2.06 × 10^5^	2954	< 20

^a)^ Apparent viscosity obtained at 0.1 s^−1^;

^b)^ Apparent viscosity obtained at 100 s^−1^.

The character of each colloid also influenced the organization of Ag_9_‐NCs within the self‐assemblies. Examination of the Col_r_ phases by SAXS measurements showed that the ribbons formed in the hexagonal and cubic phases shared quite similar lattice parameters with those observed in the lamellar phase (Figure 3i). In F127‐based system, the shoulder peak assigned to the averaged distance between adjacent Ag_9_‐NCs within the column of the Col_r_ phase was also detected (marked by the arrowhead). For ribbons formed in PEG solution, a shrunken lattice was detected with four extra peaks which could hardly assigned to the Col_r_ phase (Figure 3i, marked by stars), which indicates that the organization of Ag_9_‐NCs within the column is more complicated. Of course, the presence of a concomitant unknown phase in addition to the Col_r_ phase could not be fully excluded at the moment. PL measurements showed that the emission from the colloids of C_12_E_4_, F127, and PEG almost superpose when excited at 420 nm (Figure S21, Supporting Information). Examination of the optimized excitation on the F127‐based system (Figure S22, Supporting Information) only revealed quite a limited difference (≈10 nm smaller) compared to the C_12_E_4_‐based system. For Tyloxapol‐based system, the emission was heavily intervened by the strong blue‐emission from Tyloxapol (Figure S23, Supporting Information). Rheological measurements showed that the degree of mechanical reinforcement for the hexagonal LLC phase caused by the self‐assembly of Ag_9_‐NCs is similar to that observed in the lamellar LLC phase formed by C_12_E_4_, while it is not obvious for the cubic LLC phase formed by F127 as the mesophase itself already has an extremely large viscosity (Figure S24, Supporting Information). For the low viscous PEG solutions, the mechanical reinforcement is the most obvious. As seen from Figure 3j,k, the viscosity of the composite solution increased significantly with a much more pronounced shear‐thinning behavior, and the intrinsically only viscous sample became viscoelastic.

Further extension showed that in the LLC phase formed by an anionic surfactant (sodium dodecyl sulfate, SDS), the ribbons could form without obvious macroscopic phase separation (Figure S25, Supporting Information). However, this is not the case for cationic surfactant (cetyltrimethylammonium bromide, CTAB) where precipitation formed driven by the electrostatic attraction between negatively‐charged Ag_9_‐NCs and the positively‐charged aggregates of CTAB. It should also be noted that the usage of Ag_9_‐NCs is necessary for the formation of the ribbons. In control experiment, no ribbon was found when Ag_9_‐NCs were replaced by AgNO_3_, even with a higher content (5.0 wt.%, Figure S26, Supporting Information).

**Scheme 2:**
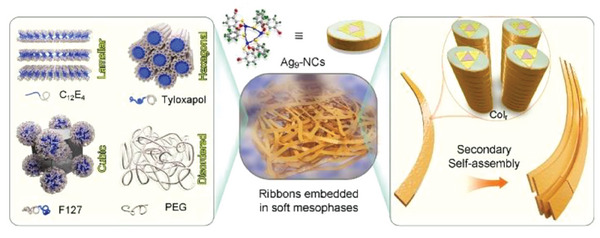
Illustration of the composite materials where ribbons from self‐assembly of Ag_9_‐NCs are embedded in various soft mesophases. The ribbon contains highly ordered Ag_9_‐NCs organized in a Col_r_ phase, which can undergo secondary self‐assembly into bundles.

Thus, our results showed that by introducing Ag_9_‐NCs into nonionic as well as anionic crowded colloids, formation of ribbons will be triggered regardless of the spatial organization of the colloids. When trapped within these colloidal matrixes, the originally soluble Ag_9_‐NCs will be pushed into the confined water channels, leading to an abrupt increase in their local concentration. The hydrophilic polyether will compete with the peripheral carboxylic groups of Ag_9_‐NCs in hydration. These two effects synergistically triggered the crystallization of Ag_9_‐NCs followed by an axial growth, leading to the formation of 1D stacks of Ag_9_‐NCs which further assemble parallelly into ribbons. During this process, the peripheral ligands of Ag_9_‐NCs were forced to adopt an uneven distribution due to the crowded microenvironment, making the originally circular cross‐section of Ag_9_‐NCs (see Scheme 1a) elliptical and accounting for the rectangular organization of the stacks. As the crystallized Ag_9_‐NCs are hard and sharp with decreased hydrophilicity, they can cross the soft hydrophobic domains formed by surfactants. With the continuous growth of the ribbons, adjacent ribbons can join together to form bundles, which can undergo dissociation and branching. Finally, a 3D network is formed (for an intuitive impression, see the supporting movie). The process of the self‐assembly and models of ribbons embedded in the three LLC phases as well as the entangled PEG network are illustrated in **Scheme** **2**. Considering that the LLC phases formed by Tyloxapol and F127 have large viscoelasticity, we suppose that the space confinement will become more obvious, accounting for the easier self‐assembly of Ag_9_‐NCs. In the case of PEG, the easier formation of the ribbons might be ascribed to the more pronounced dehydration effect of the peripheral carboxylic groups of Ag_9_‐NCs induced by PEG which is fully composed of hydrophilic groups (see Table 1).

**Figure 4:**
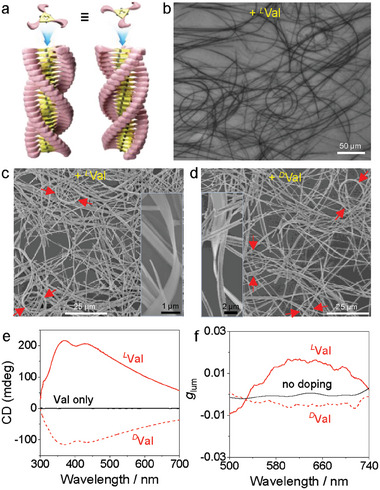
a) Proposed chirality origination from the spiral organization of Ag_9_‐NCs within the 1D stack. b) A typical optical microscopy image for the self‐assembled Ag_9_‐NCs (2.0 wt.%) in 50 wt.% C_12_E_4_ at the presence of LVal (1.5 wt.%). c) SEM image for the washed sample shown in b. d) SEM image of the sample with the same amount of Dval. Insets in c and d highlight the twisting of the nanoribbons. e) CD spectra of the two samples containing Val. For comparison, data from samples only containing Val but without Ag_9_‐NCs are also given. f) Variation of glum value as a function of wavelength for the two samples containing Val. Data with no doping is also given for comparison.

Appending specific properties, such as chirality, to the composite materials is beneficial for their practical applications. Consistent with our previous observations on self‐assembled Ag_9_‐NCs, the colloids with embedded ribbons of Ag_9_‐NCs presented in the current study also showed chiral signals despite the lack of any chiral centers of the building blocks. We have previously demonstrated that when viewed along the C_3_ axis, Ag_9_‐NC looks like a triskelion with either clockwise or anticlockwise conformation due to the referentially unidirectional arrangement of the peripheral ligands.^[^ ^26^ ^]^ During self‐assembly, the transient chirality caused by the vibration of the peripheral ligands could be fixed and amplified. As the formation of 1D stacks has been unambiguously confirmed by SAXS in the current study, another possibility for the chirality could be proposed. As seen in Scheme 1a, Ag_9_‐NC can be viewed as an analog of a C_3_‐symmetric organic molecule^[^ ^31^, ^32^, ^33^, ^34^ ^]^ where a tabular triangular prism was surrounded by organic ligands. During 1D stacking, rotation of the cluster might occur to reduce the steric hindrance (**Figure** **4**a), a process frequently observed in the self‐assembly of C_3_‐symmetric organic molecules.^[^ ^31^, ^32^, ^33^, ^34^ ^]^ These two factors discussed above account for the observed chiral signals of the composite materials, which are unfortunately random and hard to control.^[^ ^35^, ^36^, ^37^ ^]^ Taking Ag_9_‐NCs/C_12_E_4_ system as an example, during multiple measurements, both positive and negative signals were obtained from circular dichroism (CD) measurements. Recently, we showed that the uncontrolled supramolecular chirality of crystallized Ag_9_‐NCs could be manipulated by the introduction of small organic molecules with defined chirality, such as L‐ or D‐tartaric acid (TrA).^[^ ^24^ ^]^ Applying a similar strategy, herein the supramolecular chirality of the self‐assembled Ag_9_‐NCs (taking the lamellar LLC phase as an example) was tuned by Fmoc‐protected amino acid sodium salts (Val, Scheme 1a). The introduction of these chiral seeds did not destroy the morphology of the nanostructures. Imaging studies on typical samples showed that long and flexible nanostructures are present after the addition of chiral seeds (Figure 4b, Figure S27, Supporting Information). The ribbons retained their morphologies after washing and drying, where bending and twisting of the ribbons were again observed (Figure 4c, d), and magnifications on typical ribbons redisplayed the secondary self‐assembly of individual ribbons into larger ones (Figure S28, Supporting Information). CD measurements showed that enantiomeric selectivity was successful, with only one sign of the CD signal obtained for the sample containing a specific amino acid salt (Figure 4e). The regulated chirality, combined with the intrinsic photoluminescence of the ribbons, led to well‐defined CPL signals (Figure 4f), which were otherwise chaotic in the absence of the amino acid salt.

Normally, the seed effect in controlling overall chirality of self‐assembled systems contains majority rule which stands for the induced chirality by the major or lesser component respectively. During the self‐assembly of Ag_9_‐NCs into ribbons, the chiral seed could be entrapped inside the 1D matrix via physical immobilization and/or noncovalent forces. As both Ag_9_‐NC and the chiral seed bear carboxylate moieties, formation of assemblies was expected, which induced the emergence of helical ribbons with homochirality determined by the absolute chirality of the chiral seed. This seeding effect is consistent with the soldier‐and‐sergeant rule, which to date has rarely been clarified in self‐assemblies with metal NCs as the main component.^[^ ^24^ ^]^ Although further details might be needed to fully reveal the mechanisms behind the transfer and amplification of the chirality, the current study provided a novel protocol to produce chiral soft materials from achiral metal NCs, where the chiral behavior and chiroptical effects are under rational control.

## 3 Conclusion

In summary, we have presented for the first time the self‐assembly of metal NCs in crowded colloids, using the negatively charged Ag_9_‐NC as an example. The competitive hydration of the hydrophilic groups and the exclusion effect induced by the colloids triggered the CISA of Ag_9_‐NCs. The high viscosity/elasticity of the colloidal solution retarded the self‐assembly of Ag_9_‐NCs and provided the self‐assemblies resistance against gravity‐induced sedimentation, leading to the formation of ultralong ribbons with Col_r_ organization, photoluminescence, and hierarchical structure. Embedded with a network of ribbons, the mechanical strength of the colloids has been improved. By tuning with chiral seeds, the uncontrolled supramolecular chirality of the system was regulated to give homochiral signals. Combined with the AIE of Ag_9_‐NCs, CPL was achieved for the composite materials. Current study deepens our understanding of the self‐assembly of metal NCs, and the marriage between cluster science and colloid science opens a new frontier for engineering hard/soft hybrid materials with potential applications in fields of chiral optics, chiral separation, and chiral catalysis.

## Conflict of Interest

The authors declare no conflict of interest.

## Supporting information

Supporting Information

Supplemental Movie 1

## Data Availability

The data that support the findings of this study are available from the corresponding author upon reasonable request.
