# Supplementary material for: Self‐Assembly of Atomically Precise Silver Nanoclusters in Crowded Colloids into Ultra‐Long Ribbons with Tunable Supramolecular Chirality
Source: Adv Sci (Weinh). 2023 Nov 20;11(1):2305102. doi: 10.1002/advs.202305102 (PMC10767393; doi:10.1002/advs.202305102)
Supplement: Supplementary file 1 — Supporting Information [file ADVS-11-2305102-s002.pdf]

ADVANCED SCIENCE
Open Access

Supporting Information

for *Adv. Sci.*, DOI 10.1002/advs.202305102

Self-Assembly of Atomically Precise Silver Nanoclusters in Crowded Colloids into Ultra-Long Ribbons with Tunable Supramolecular Chirality

Wenjuan Wang, Tong Liu, Ting Zhao, Di Sun, Hongguang Li, Pengyao Xing* and Xia Xin**

Supporting Information

1. Experimental section

1.1 Chemicals and Materials

Silver nitrate ($AgNO_3$, AR), 2-mercaptobenzoic acid (H_2mba, AR), ammonium hydroxide ($NH_3 \cdot H_2O$, aqueous solution, 25-28 wt%), Pluronic F127 (F127, AR), tetraethyleneglycol monododecyl ether ($C_{12}E_4$, AR) and Tyloxapol (Tyl, AR) were obtained from Sigma. Polyethylene glycol (PEG) used was purchased from Tianjin Kemiou Chemical Reagent Co., Ltd. The trade name given by the manufacturer is PEG 20000, which is claimed to contain ~386 repeating unit on average. Our measurements gave a Mn of 20989 and a Mw of 24587, which yielded a polydispersity index (PDI) of ~1.17. Sodium dodecyl sulphate (SDS, 99%), cetyltrimethyl ammonium bromide (CTAB, 99%) and N-butanol (NBA, AR) was purchased from Sinopharm Chemical Reagent Co., Ltd. Fmoc-functionalized valine (Fmoc-^{L}Val-OH and Fmoc-^{D}Val-OH, AR) and Fmoc-functionalized *D*-alanine (Fmoc-^{D}Ala-OH, AR) were purchased from Heowns. Fmoc-functionalized *L*-alanine (Fmoc-^{L}Ala-OH, AR) was purchased from Aladdin. Sodium hydroxide (NaOH, AR) was obtained from Macklin. All the chemicals were used without further purification. Ultrapure water used in the experiments with a resistivity of 18.25 MΩ cm was obtained using a UPH-IV ultrapure water purifier (China).

Ag_9-NCs were prepared following the established procedures.[1-3] In a typical experiment, $AgNO_3$ (1 mmol, 170 mg) and H_2mba (1 mmol, 155 mg) were dispersed in 6 mL water. The mixture was sonicated in a KQ5200DE instrument (80 W, Kun Shan Ultrasonic Instruments Co.) for 20 min, during which 0.5 mL of $NH_3 \cdot H_2O$ was added. A yellow clear solution of Ag_9-NCs was obtained, whose concentration was calculated to be ~17.1 $mmol \cdot L^{-1}$ (~4.0 wt%).

1.2 Instruments and methods

1.2.1 Imaging study

Optical microscopy and polarized optical microscopy (POM) observations were performed with a “Axio SCOP. A1” A Pol (ZEISS, Jena, Germany) microscope fitted with AxioVision SE64. Confocal laser scan microscopy (CLSM) observations were performed using an inverted microscope (model IX81, Leica, Germany) equipped with a high-numerical-aperture 60 oil-immersed objective lens. Field emission scanning electron microscopy (FE-SEM) observations were carried out on JSM-6700F (JEOL Ltd., Tokyo, Japan) under 10 kV. Transmission electron microscopy (TEM) observations were carried out on a JEM-100CX II (JEOL Ltd., Tokyo, Japan) operating at 80 kV with a Gatan multiscan CCD recording the images. Atomic force microscopy

(AFM) observations were carried out in a tapping mode operating with Bioscope Resolve (Bruker, USA) at a scan frequency of 1.5 Hz.

1.2.2 Structural determination

X-Ray photoelectron spectroscopy (XPS, on the washed sample) was operated on ESCALAB 250 X-ray photoelectron spectrometer with a monochromatized Al Ka X-ray source (1486.71 eV). X-ray diffraction (XRD) spectra were acquired using a D8 ADVANCE (Bruker, Germany) diffractometer equipped with a Cu Kα radiation source and a graphite monochromator. Fourier transform infrared (FTIR) spectra were obtained using KBr pellets on a Tensor II spectrometer (Bruker, Germany). Small angle X-ray scattering (SAXS) measurements are performed using an Anton-Paar SAX Sess mc^2 system (Austria) with Ni-filtered Cu Kα radiation (1.54 Å) operating at 50 kV and 40 mA.

1.2.3 Optical measurements

UV-vis spectra were recorded on a UV-vis-NIR spectrometer (Cary 5000, Agilent, USA), with samples prepared in between quartz wafers. The fluorescence measurements were performed on a LS-55 spectrofluorometer (FluoroMax-4, Horiba). The fluorescence lifetimes were obtained from an Edinburgh Instruments FLS920 luminescence spectrometer (xenon lamp, 450 W).

1.2.4 Rheological measurements

Rheological measurements were carried out in oscillatory mode on a HAAKE RS6000 rheometer with a cone-plate system (Ti, diameter, 35 mm; cone angle, 1º). An amplitude sweep at a fixed frequency of 1 Hz was performed prior to the following frequency sweep in order to ensure the selected stress was in the linear viscoelastic region. The temperature was maintained at 25.0 ± 0.1 ºC with the help of a cyclic water bath. CD and CPL were carried on Chirascan V100 instrument (Appplied Photophysics, UK). Samples were prepared between quartz wafers before testing.

1.3 Sample preparation for general characterizations

To gain a comprehensive understanding of the nanoribbons, samples were characterized both *in situ* and after suitable pretreatments. These mainly includes three parts:

1.3.1 In-situ characterization

Samples were characterized directly on the as-prepared samples, i.e., the nanoribbons embedded in the highly-condensed colloids. In brief, to a vial was added desired amount of surfactant (F127, $C_{12}E_4$ or Tyr) or PEG, followed by the addition of the stock solution of Ag_9-NCs and water. The total mass of each sample is 1 g. After being fully mixed using a stirring rod, the samples were then placed in a incubator at 25 °C before characterization.

Characterizations performed with such *in situ* prepared samples include i) optical microscopy,

POM and CLSM observations; ii) all of the optical measurements; iii) the rheological measurements.

1.3.2 The powders after washing

Samples were also washed to remove the soluble "impurities". For this purpose, samples prepared from Pluronic F127 were washed multiple times with water. We noticed that the "impurities" for the samples from $C_{12}E_4$ and Tyl could not be fully removed by water. These samples were washed multiple times with NBA, which acted better than water. After that, the lyophilized samples (powders) were subjected to XPS, XRD and FTIR (KBr pellets) measurements.

For TEM, FE-SEM and AFM observations, the powders were suspended in water, from which one drop was dropped onto a copper grid or a silicon wafer followed by being dried with an infrared lamp for 30 min.

For SAXS, measurements were performed both on the unwashed, as-prepared samples and on the powders.

1.4 Sample preparation for chirality-related studies

Stock solutions of amino acid sodium salts, i.e., Fmoc-^{L}Val-ONa/Fmoc-^{D}Val-ONa (0.5 mmol·L^{-1}, 18.07 wt%) and Fmoc-^{L}Ala-ONa/Fmoc-^{D}Ala-ONa (0.5 mmol·L^{-1}, 16.67 wt%) were prepared by mixing equimolar amount of each amino acid and NaOH in water. For the preparation of samples containing amino acid sodium salts, desired amounts of $C_{12}E_4$, stock solutions of Ag_9-NCs and each amino acid sodium salt were added to a vial. The sample has a final composition of 50 wt% $C_{12}E_4$, 2.0 wt% Ag_9-NCs and 1.5 wt% amino acid sodium salt. After being fully mixed using a stirring rod, the samples were then placed in an incubator at 25 °C before characterization.

Data of CD and CPL were recorded on a Chirascan V100 circular dichroism spectrometer (Applied Photophysics, British). The as-prepared samples were prepared between quartz wafers, and measurements were performed using reflection mode for CPL and transmission mode for CD.

1.5 Structural assignment of the ribbon by SAXS

The internal structure of the ribbon was assigned based on data from SAXS. Columnar mesophase Col_{r} with rectangular geometry follows the following equation:

$$\frac{1}{d_{hk0}^2} = \frac{h^2}{a_r^2} + \frac{k^2}{b_r^2}$$

The first two peaks from the self-assemblies of Ag_9-NCs, which locate between 3 nm-1 and 6 nm-1, are assigned to the (11) and (20) planes of the Col_{r} phase. From the values of d and the plane

indexes, a_r and b_r could be obtained.

The validity of the Col_r phase was then checked by the positions of (30) and (40) planes, which fits quite well in all the cases.

2. Additional Data

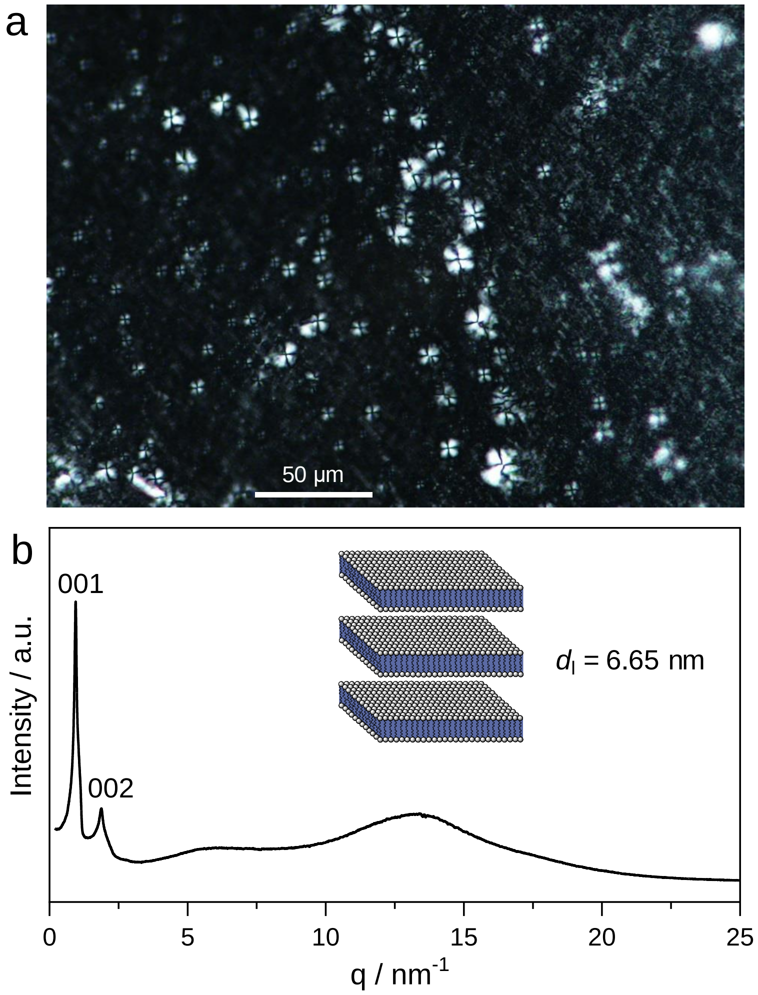

Figure S1. A typical POM image (a) and the result of SAXS (b) for the aqueous solution of $C_{12}E_4$ (50 wt%). Inset of b is illustration of the lamellar phase. The first two planes (001, 002) are marked and the interlamellar spacing (d) is given.

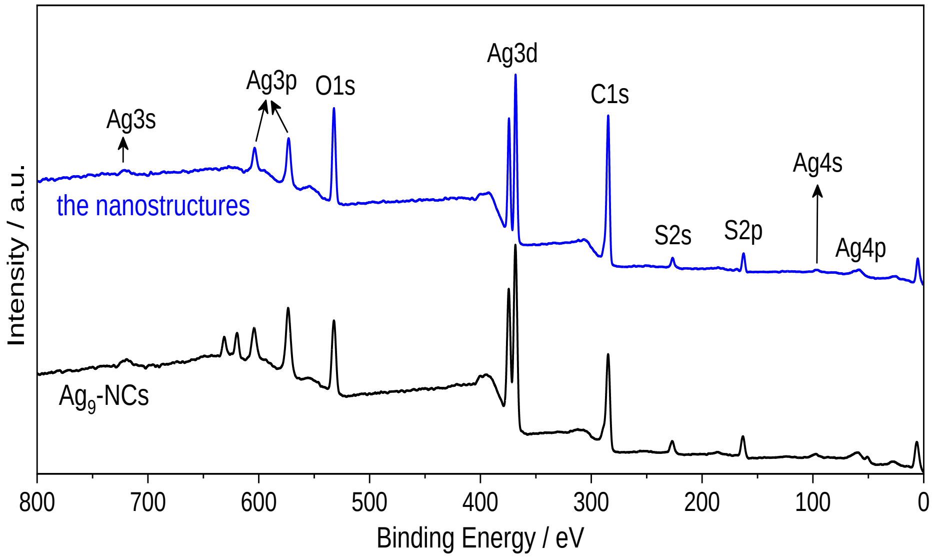

Figure S2. XPS curve of the washed nanostructures self-assembled from Ag_9-NCs (2.0 wt%) in the lamellar LC phase of $C_{12}E_4$ (50 wt%). For comparison, the curve of the lyophilized Ag_9-NCs is also given.

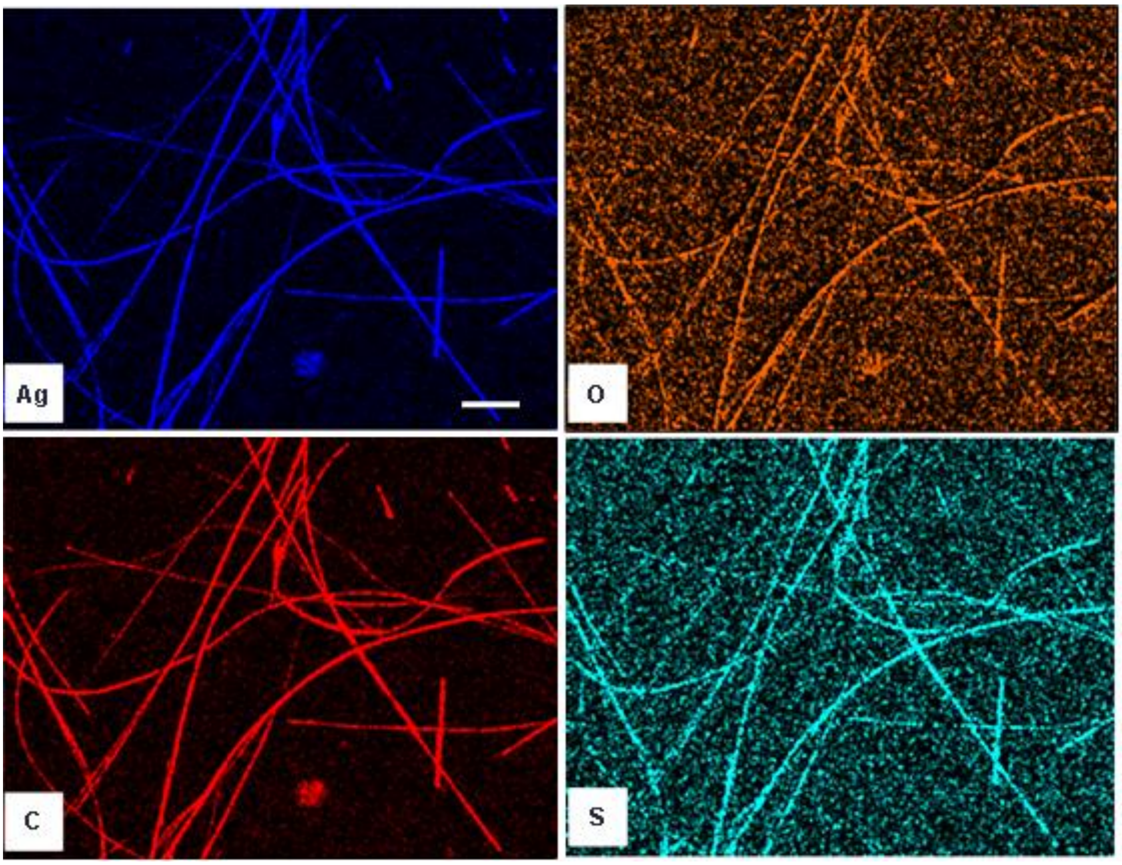

Figure S3. EDS mapping of the washed self-assemblies from Ag_9-NCs (2.0 wt%) in the lamellar LC phase of $C_{12}E_4$ (50 wt%). The scale bar corresponds to 30 μm.

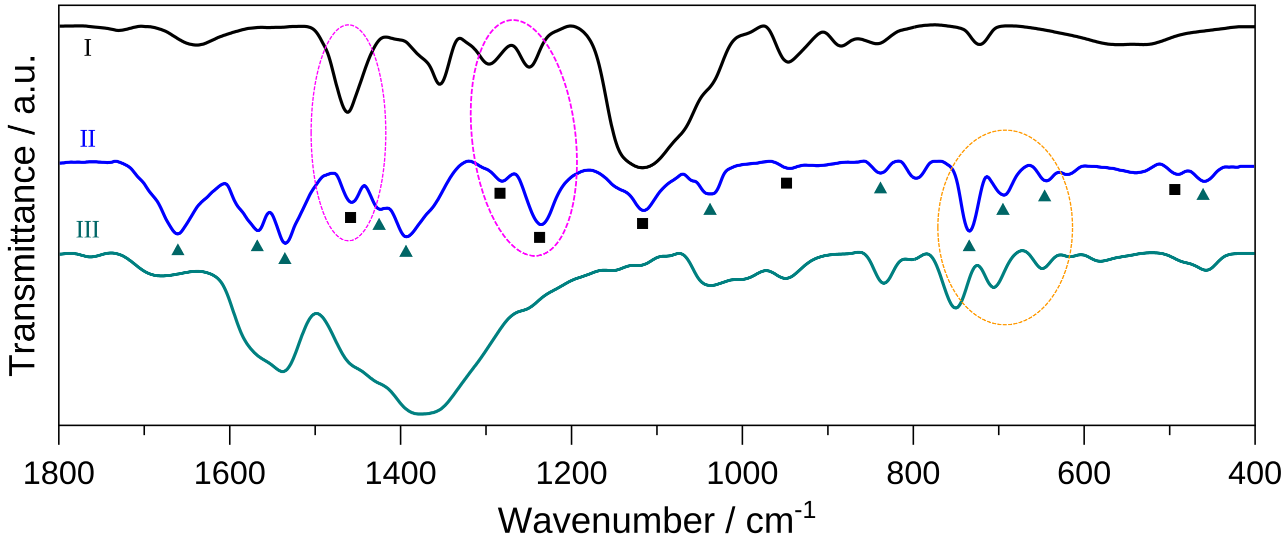

Figure S4. FTIR spectra of 50 wt% $C_{12}E_4$ aqueous solution (curve I), the washed self-assemblies of 2.0 wt% Ag_9-NCs (curve II) and the lyophilized Ag_9-NCs (curve III).

Note: *The spectrum of the washed self-assemblies combines the feature of both the surfactant (the squares) and lyophilized* Ag_9*-NCs (the triangles). Meanwhile, peak shift was observed in both cases (the dashed ellipses).*

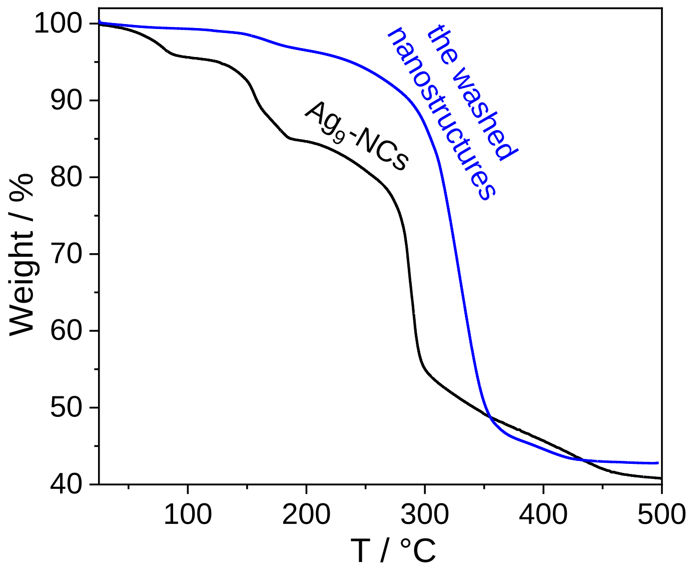

Figure S5. TGA curve of the washed ribbons self-assembled from Ag_9-NCs (2.0 wt%) in the lamellar phase of $C_{12}E_4$ (50 wt%). For comparison, the curve of the lyophilized Ag_9-NCs is also given.

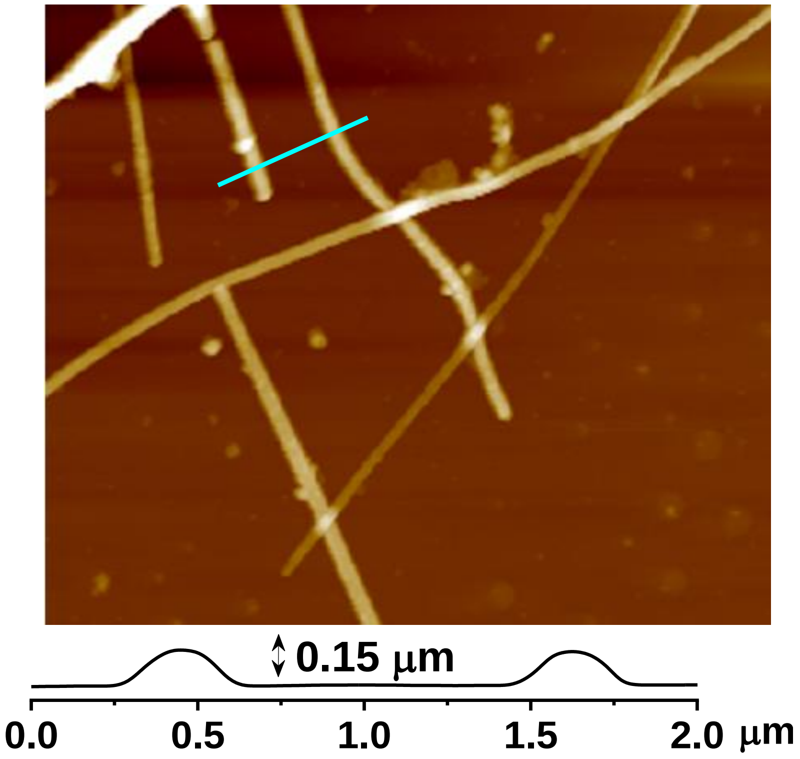

Figure S6. AFM image of the washed self-assemblies of Ag_9-NCs (2.0 wt%) formed in the lamellar LC phase of $C_{12}E_4$ (50 wt%). Bottom is the height profile along the straight line inside the image.

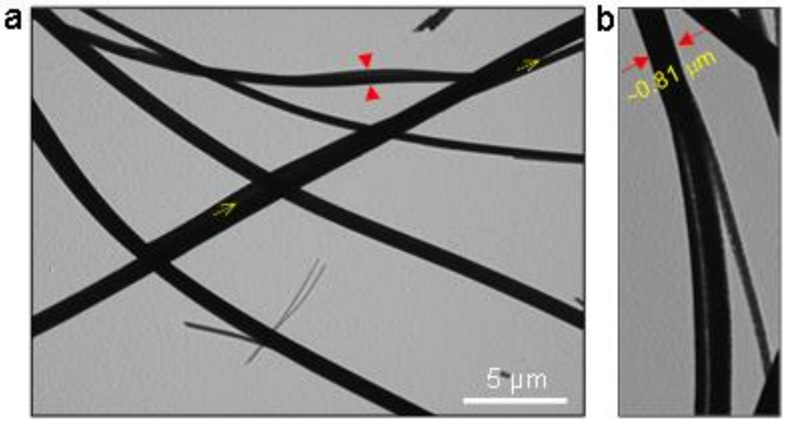

Figure S7. a) A typical TEM image of the washed self-assemblies of Ag_9-NCs (2.0 wt%) formed in the lamellar LC phase of $C_{12}E_4$ (50 wt%). The change of the width of the nanostructure in the upper is clear, which is caused by the twisting of the nanoribbon. The widest point is indicated by the arrow heads. The thick nanoribbon is composed of several smaller subunits, and the cracks are marked by the arrows. b) A TEM image highlights the divarication of a big nanoribbon.

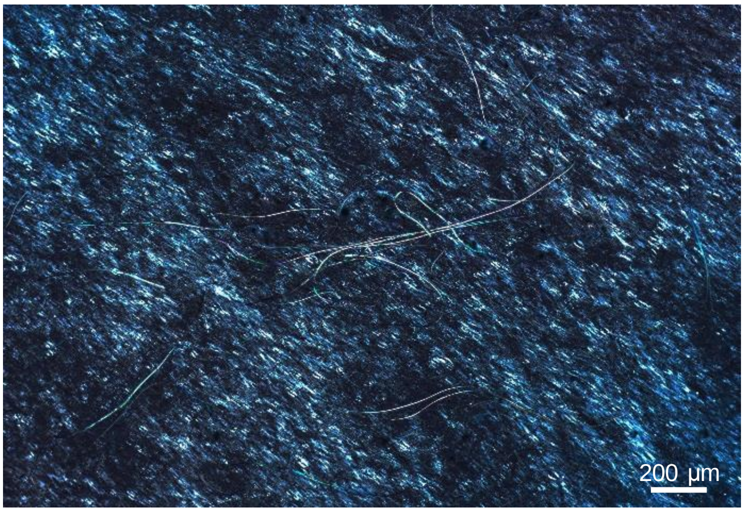

Figure S8. A typical POM image of 50 wt% $C_{12}E_4$ containing 1.5 wt% Ag_9-NCs. Formation of one-dimensional nanostructures was confirmed, despite of the low number density.

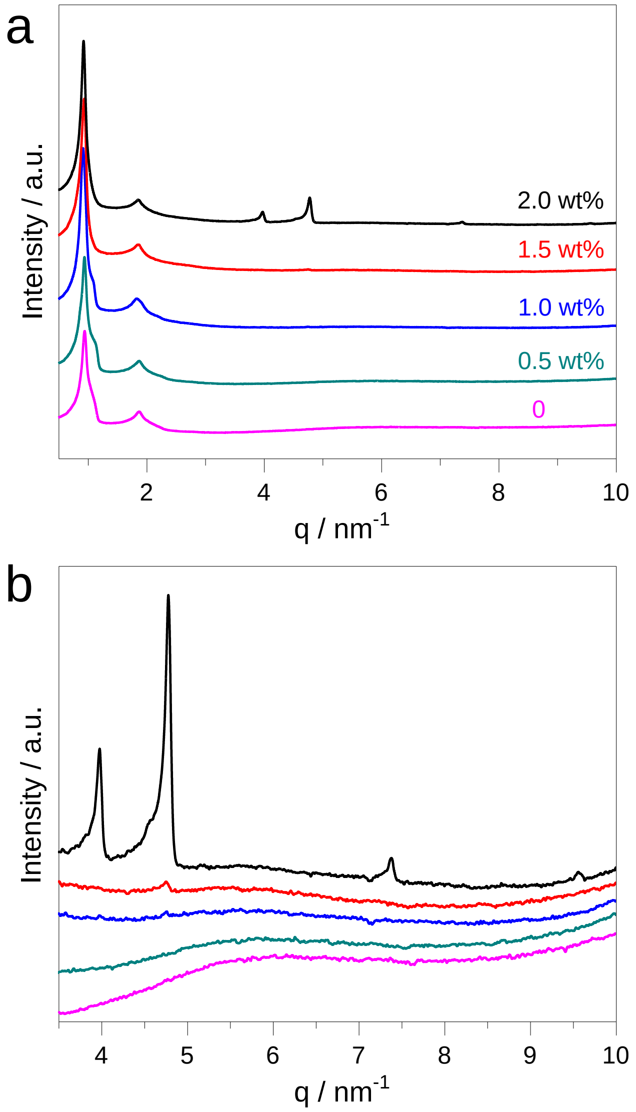

Figure S9. SAXS patterns of 50 wt% $C_{12}E_4$ containing varying amount of Ag_9-NCs as indicated. a) In the q range of 0.5-10 nm^{-1}. b) In the q range of 3.5-10 nm^{-1} which highlights the peaks from the Col_r phase, which became almost undetectable when the amount of Ag_9-NCs decreased to 1.5 wt%.

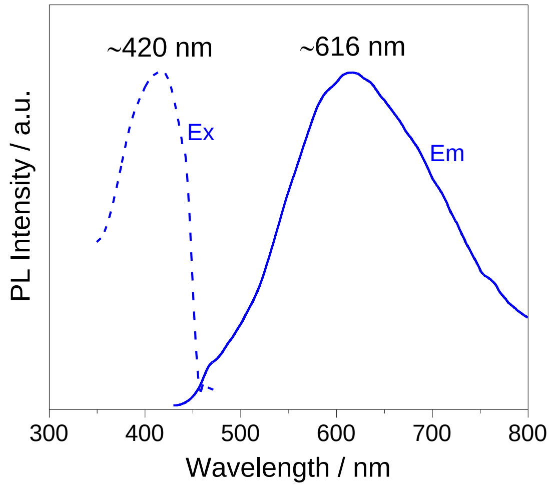

Figure S10. The excitation curve (dashed line) and the emission curve (solid line, excited at 420 nm) of the self-assembled Ag_9-NCs (2.0 wt%) in $C_{12}E_4$ (50 wt%).

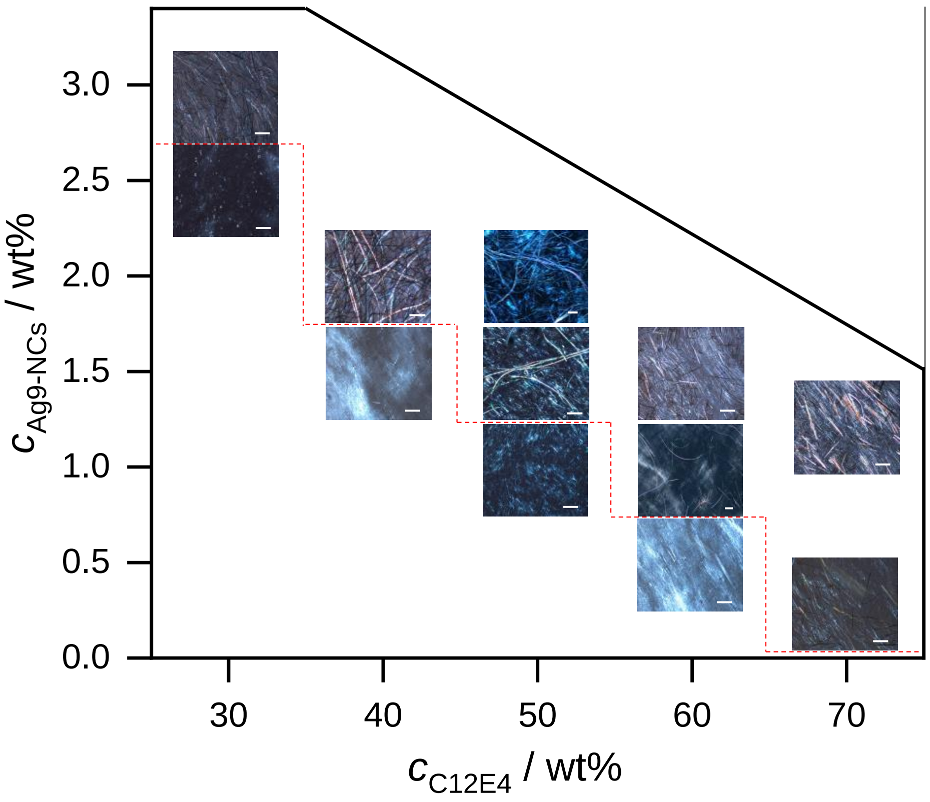

Figure S11. A sample matrix for $C_{12}E_4$/Ag_9-NCs/H_2O ternary system, with typical POM images shown inset. The dashed line denotes the phase boundary, with the upper one obviously containing the nanoribbons. In the bottom images, nanoribbons are hardly seen. The scale bar in each image corresponds to 100 μm.

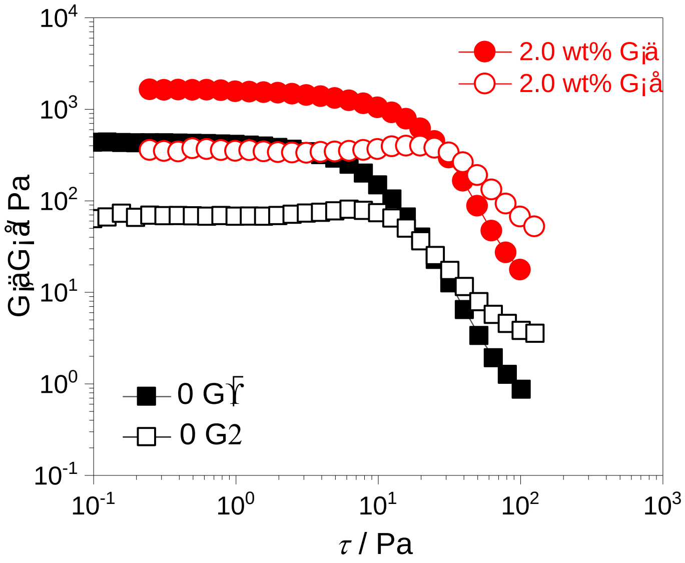

Figure S12. Variations of the elastic modulus G′ and viscous modulus G″ as a function of the applied shear stress τ for Ag_9-NCs (2.0 wt%) in $C_{12}E_4$ (50 wt%), recorded at a fixed frequency of 1.0 Hz (6.28 rad·s^{-1}). For comparison, data from the sample without Ag_9-NCs are also given.

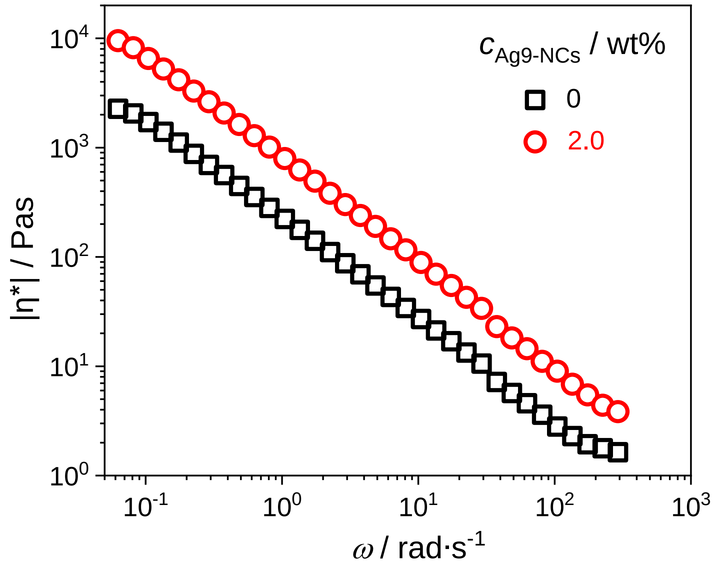

Figure S13. Variations of the complex viscosity |η*| as a function of the angular frequency ω for Ag_9-NCs (2.0 wt%) in $C_{12}E_4$ (50 wt%). For comparison, data from the sample without Ag_9-NCs are also given.

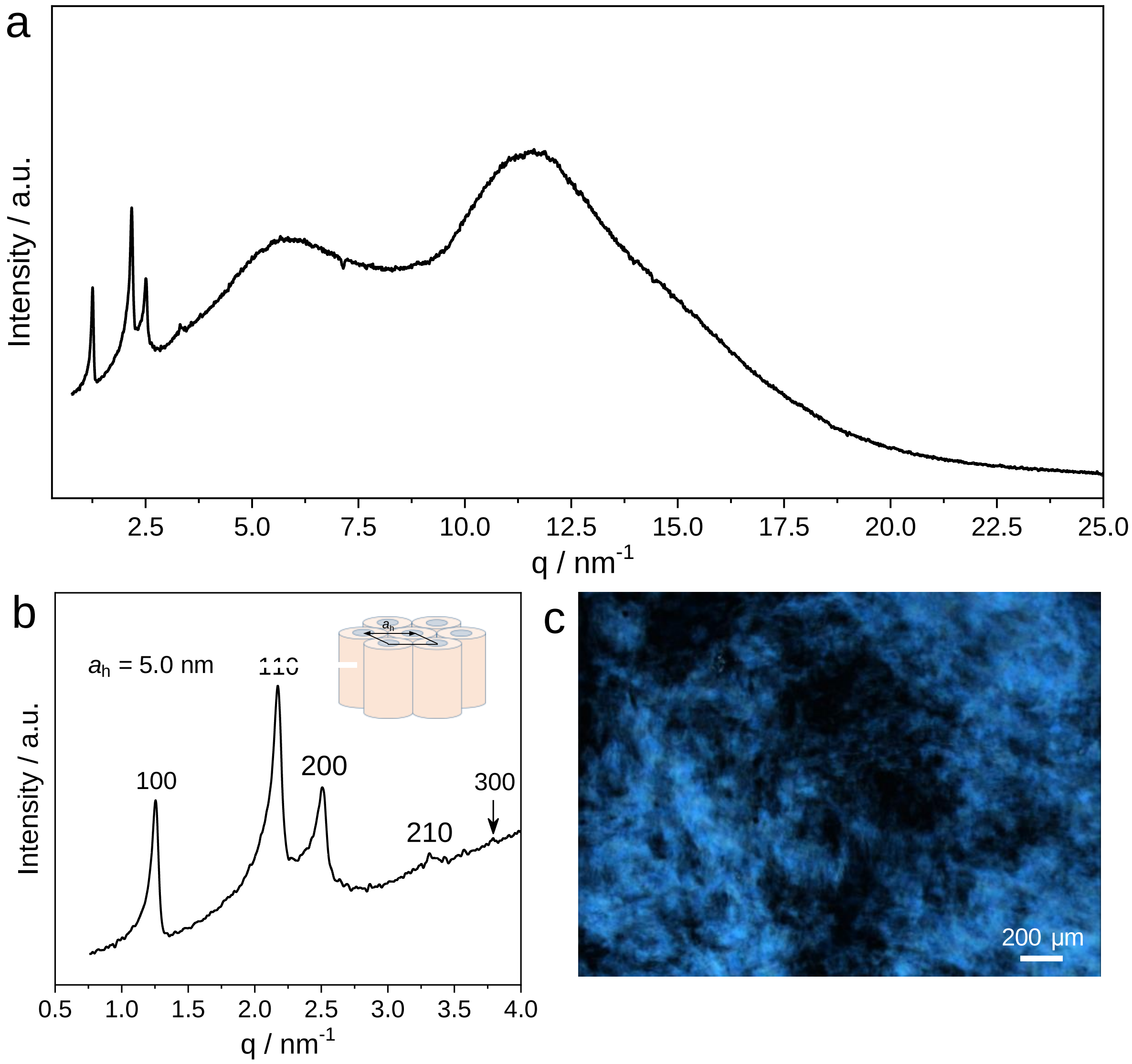

Figure S14. a) SAXS curve of aqueous solution of Tyloxapol with a concentration of 50 wt%. b) The enlarged SAXS curve within the small q region, which was indexed to be a hexagonal phase (an illustration of the structure is given inset). c) POM image of the sample.

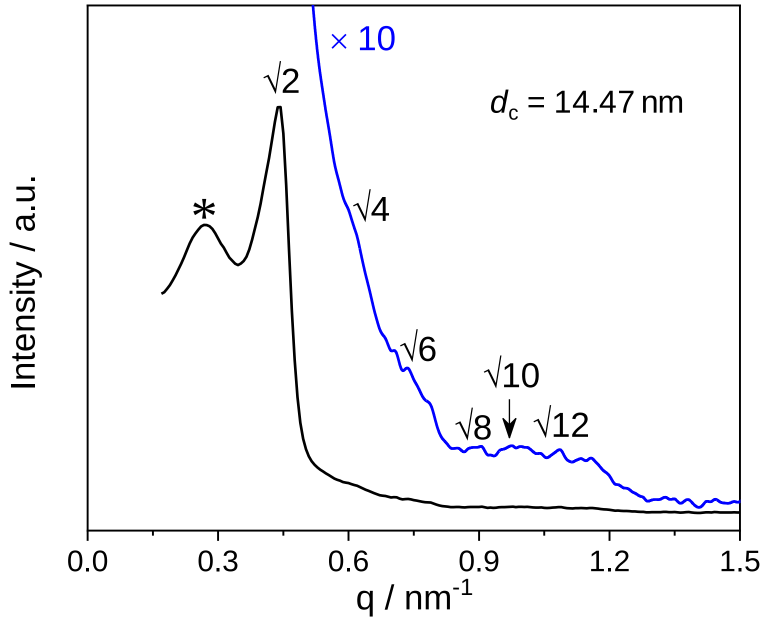

Figure S15. SAXS curve of aqueous solution of F127 with a concentration of 50 wt%, which was indexed to be a body-centered cubic phase.

Data notion: The broad peak marked by the star is caused by the less ordered F127 micelles involved in the solution, which could be partially ascribed to the polydispersity of the polymer. Another possibility is that the incubation time is not enough for the phase to get equilibrium. This is especially true for the extremely viscous cubic phase in current study, which needs a long time for phase equilibrium.

In current study, to give enough time to reach phase equilibrium is not the necessity, as our main purpose is to obtain long, flexible aggregates of Ag_9-NCs, and to make comparison between the effects of different colloids.

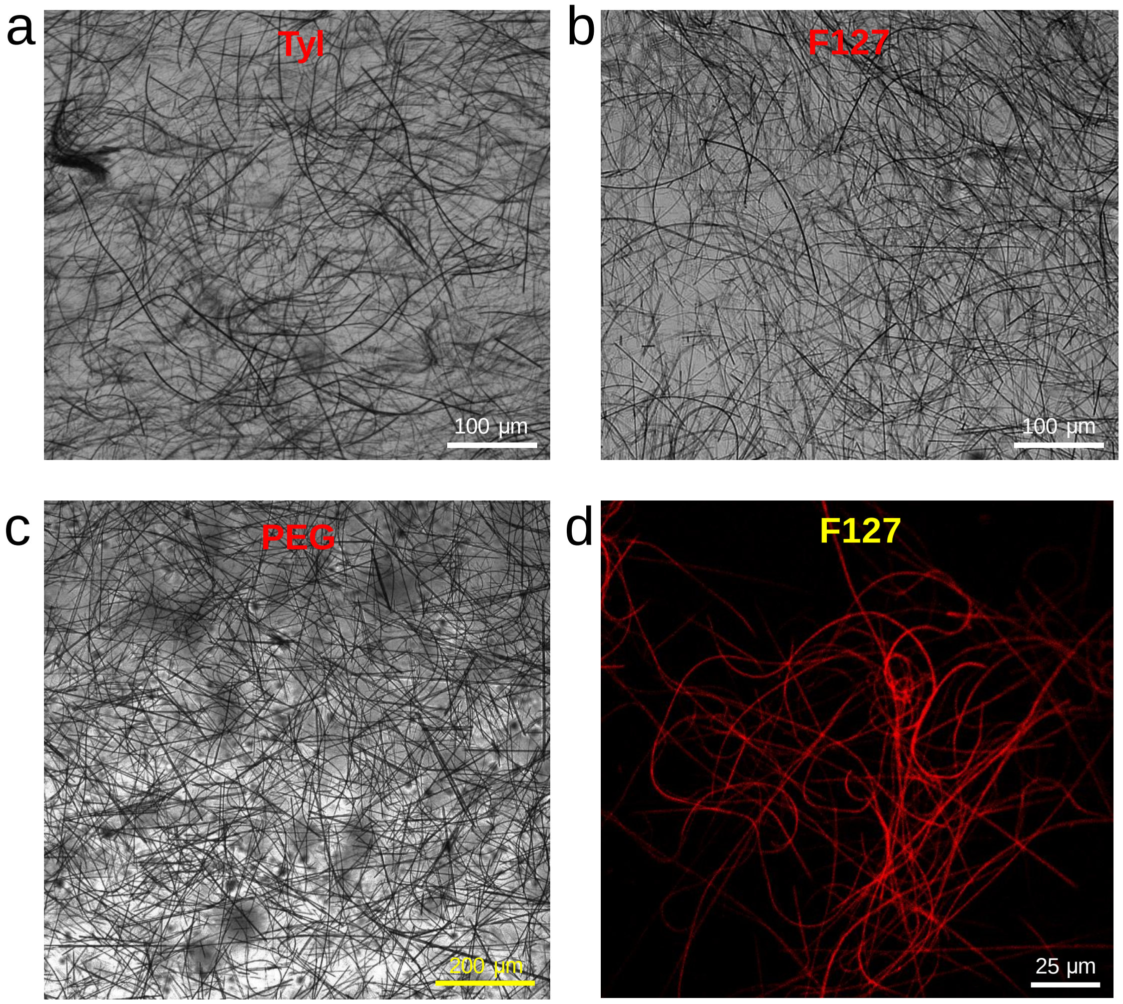

Figure S16. Additional POM (a-c) and CLSM (d) images for the nanostructures formed by Ag_9-NCs (2.0 wt%) in aqueous solutions of different polyether (50 wt%).

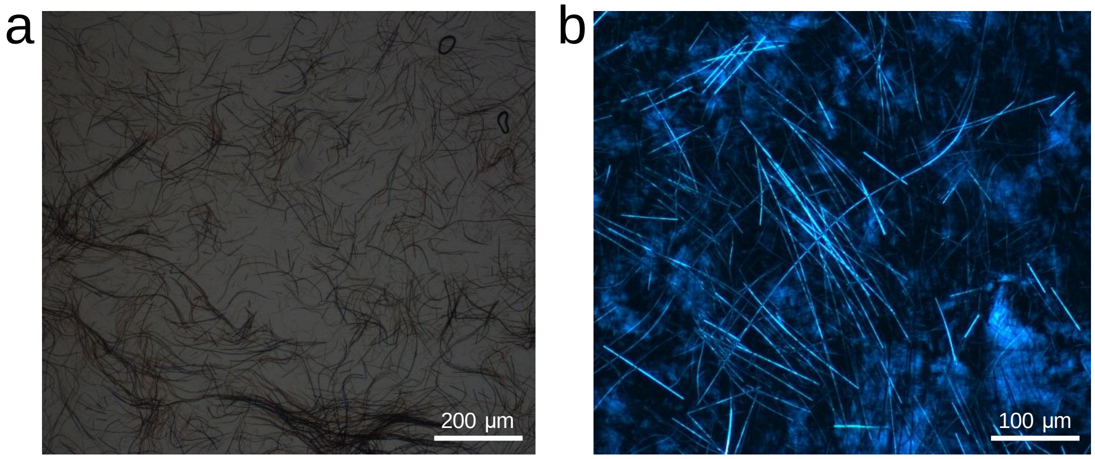

Figure S17. Optical (a) and POM (b) images for the self-assemblies of Ag_9-NCs (1.5 wt%) in the hexagonal LC phase of Tyloxapol (50 wt%).

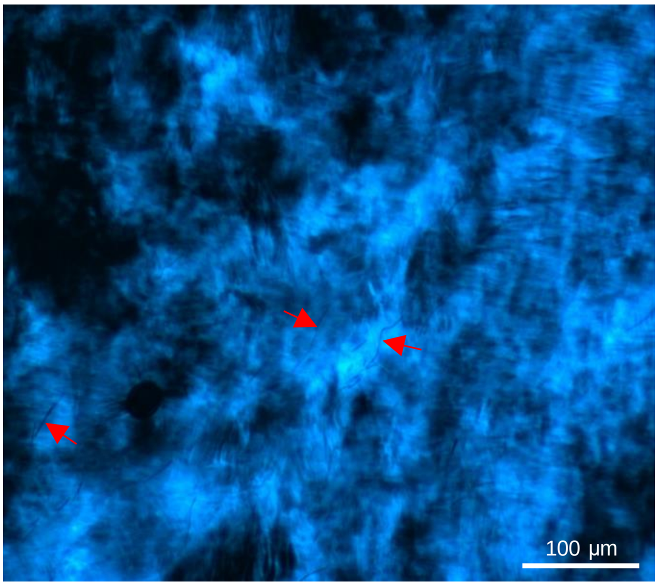

Figure S18. A typical POM image obtained from the sample containing 1.0 wt% Ag_9-NCs and 50 wt% Tyloxapol. Despite of the relatively low concentration, self-assembly of Ag_9-NCs is confirmed. Some elongated self-assemblies are marked by the arrows.

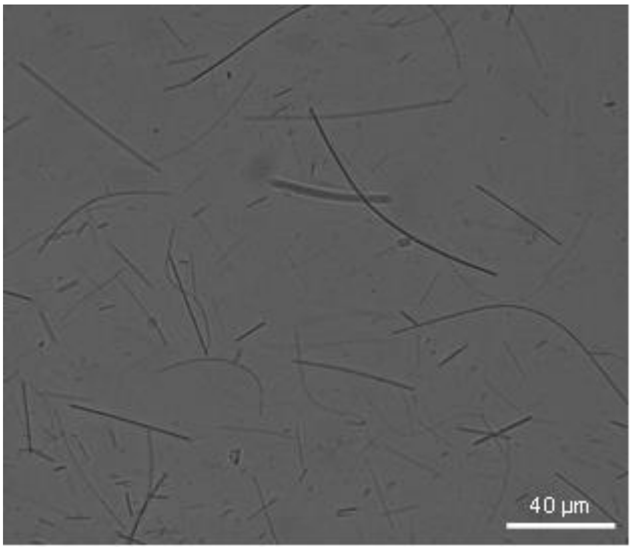

Figure S19. A typical optical microscopy image showing the presence of self-assembled Ag_9-NCs (2.0 wt%) in aqueous solution of F127 with a concentration of 30 wt%.

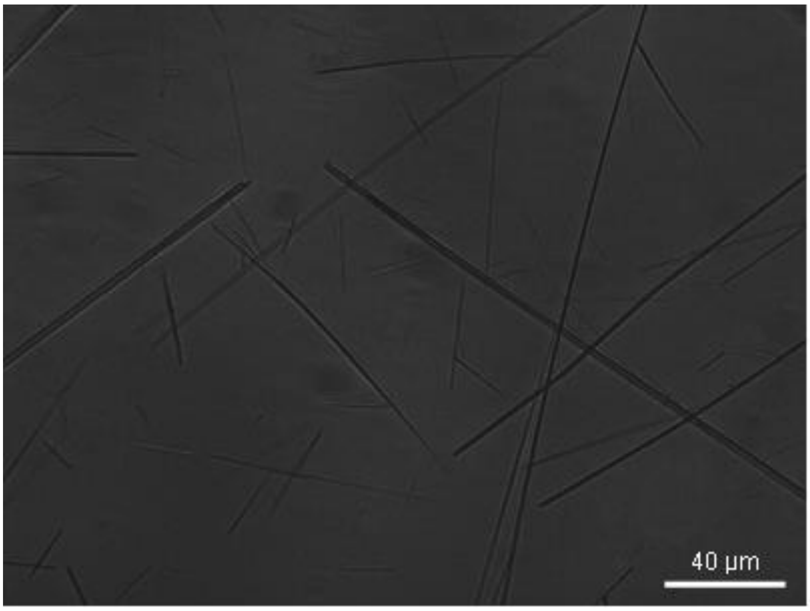

Figure S20. A typical optical microscopy image showing the presence of self-assembled Ag_9-NCs (2.0 wt%) in aqueous solution of PEG with a concentration of 40 wt%.

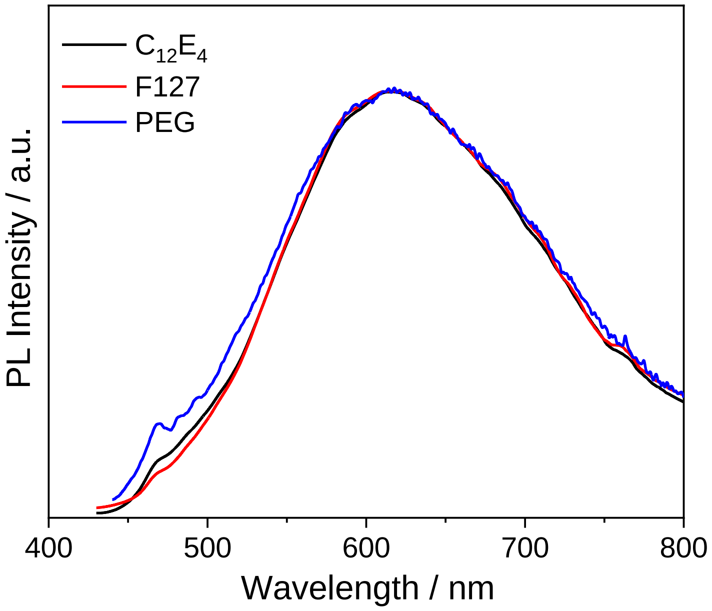

Figure S21. Normalized emission (excited at 420 nm) of the three LC phases (50 wt%) as indicated, each contains 2.0 wt% Ag_9-NCs.

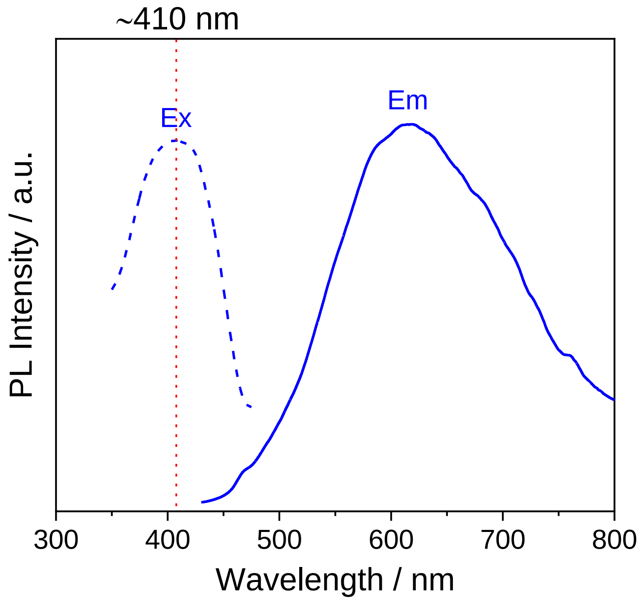

Figure S22. The excitation curve (dashed line) and the emission curve (solid line, excited at 410 nm) for Ag_9-NCs (2.0 wt%) in F127 (50 wt%).

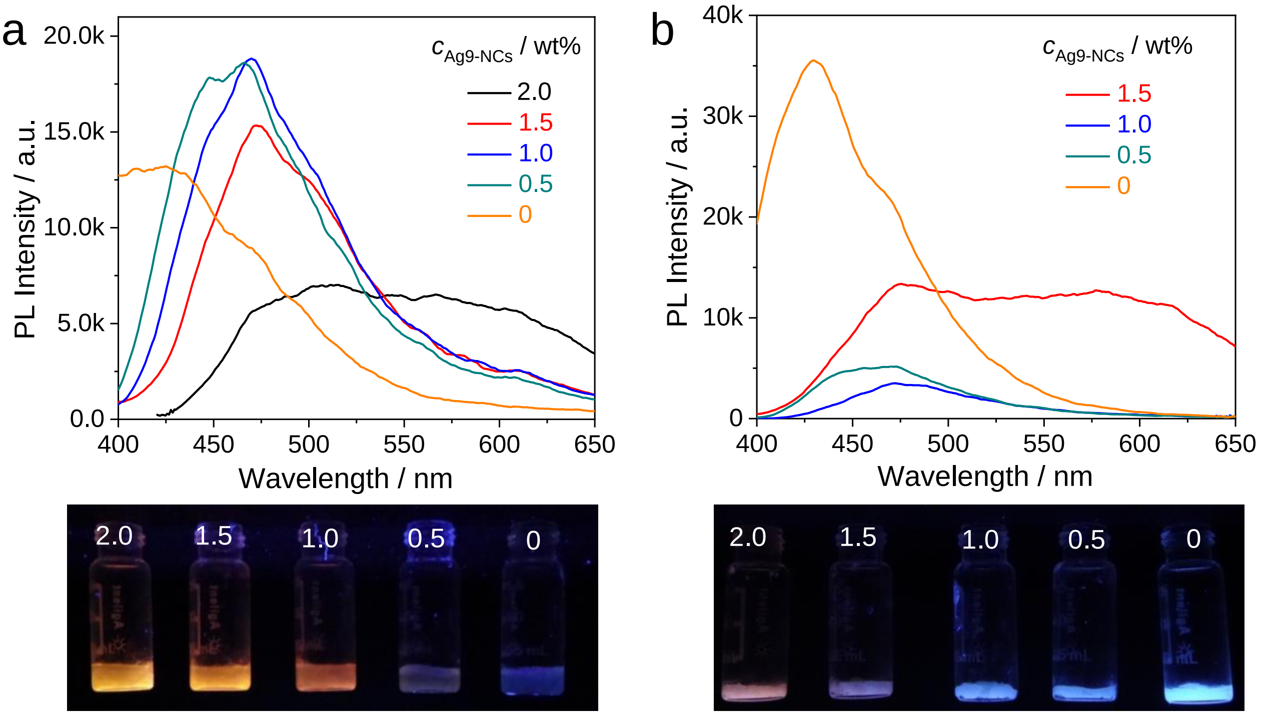

Figure S23. Comparison of the emission curves (excited at 365 nm) of the self-assembled Ag_9-NCs embedded in the lamellar LC phases formed by $C_{12}E_4$ (a) and the hexagonal LC phase formed by Tyloxapol (b). In both cases, the concentration of the surfactant is fixed at 50 wt% and the contents of Ag_9-NCs are shown insets. In the bottom, photos of the samples under 365 nm UV irradiation are presented. For the system of Tyloxapol, the broadening of the emission occurred when $c_{Ag9\text{-}NCs}$ reaches 1.5 wt%, which is smaller than that of $C_{12}E_4$ system (2.0 wt%). The strong blue emission of Tyloxapol has been reported by us previously,[4] while the weak emission of $C_{12}E_4$ could be ascribed to the nonconventional luminescence[5] from the clustering of the electron-rich atoms (oxygen in this case).

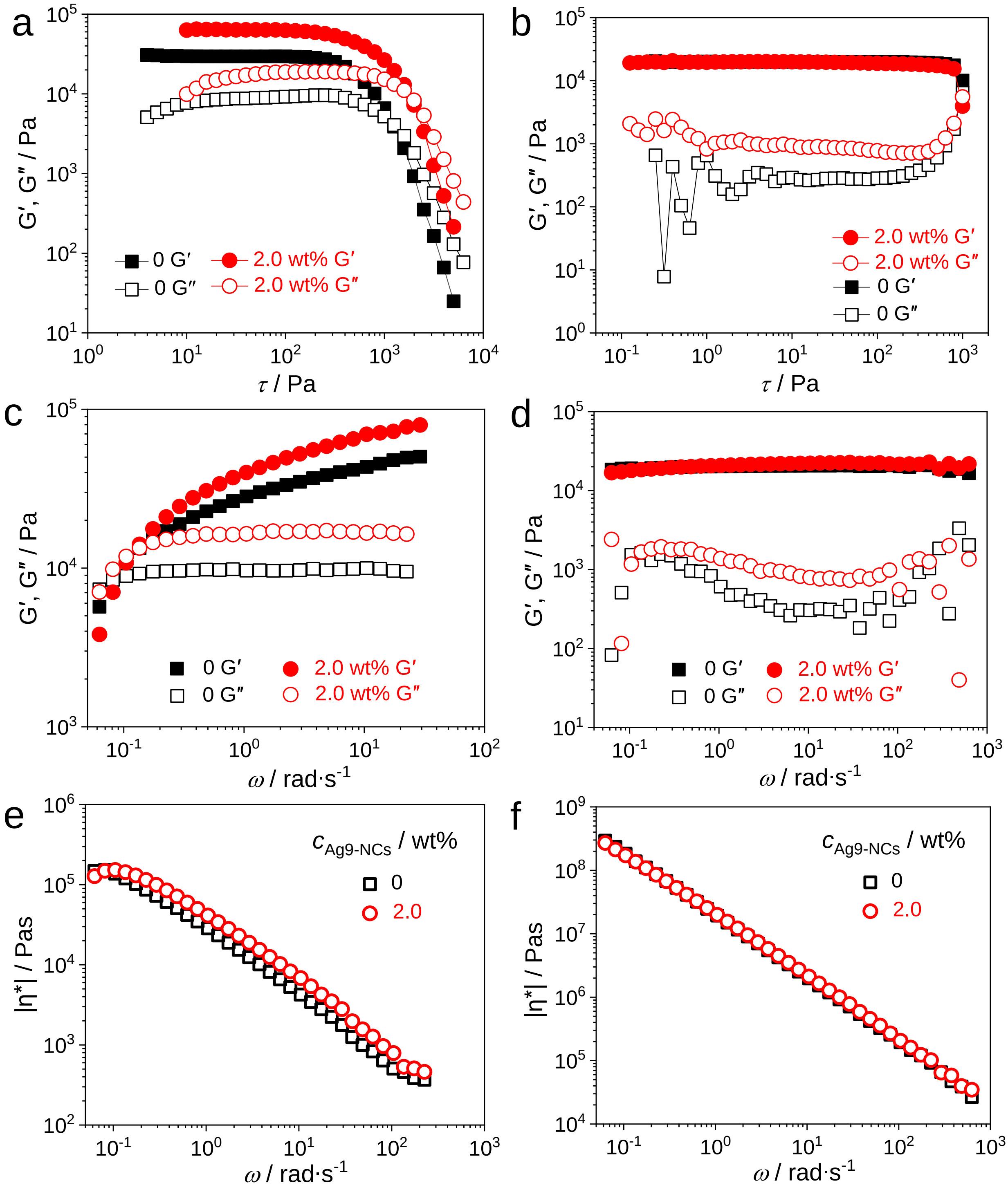

Figure S24. Variations of a,b) G′, G″ as a function of the applied shear stress at a fixed frequency of 1.0 Hz, c,d) G′, G″ as a function of ω and e,f) |η*| as a function of ω for 2.0 wt% of Ag_9-NCs in 50 wt% of Tyloxapol (a, c, e) and F127 (b, d, f).

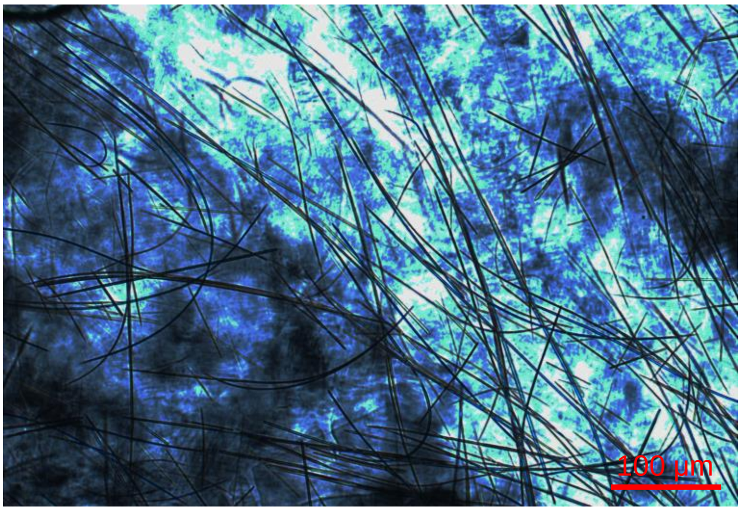

Figure S25. POM image for 50 wt% of SDS aqueous solution containing 2.0 wt% Ag_9-NCs.

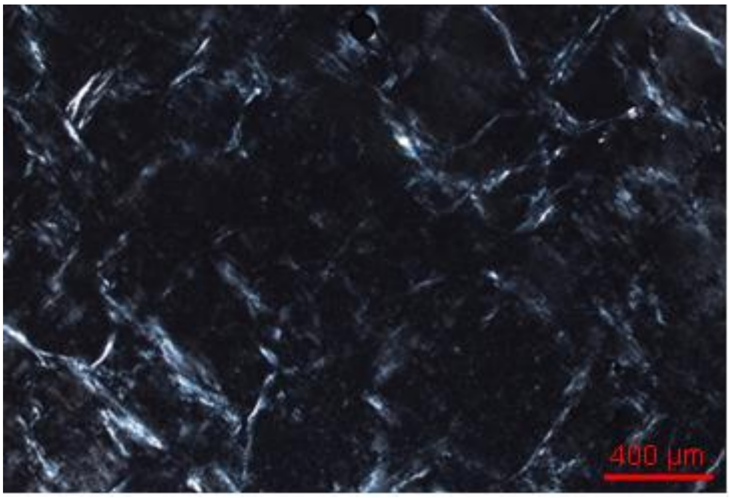

Figure S26. POM image for the lamellar LLC phase of $C_{12}E_4$ (50 wt%) containing 5.0 wt% $AgNO_3$.

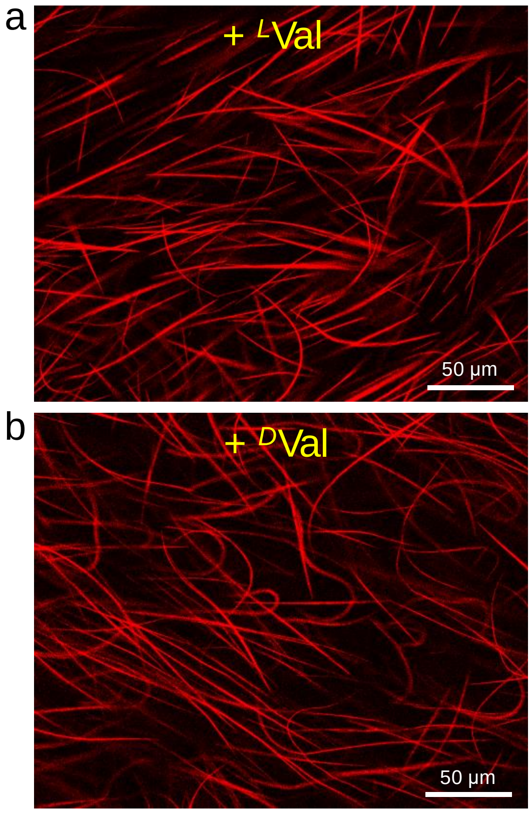

Figure S27. CLSM images of self-assembled Ag_9-NCs (2.0 wt%) in 50 wt% $C_{12}E_4$ at the presence of Val (1.5 wt%).

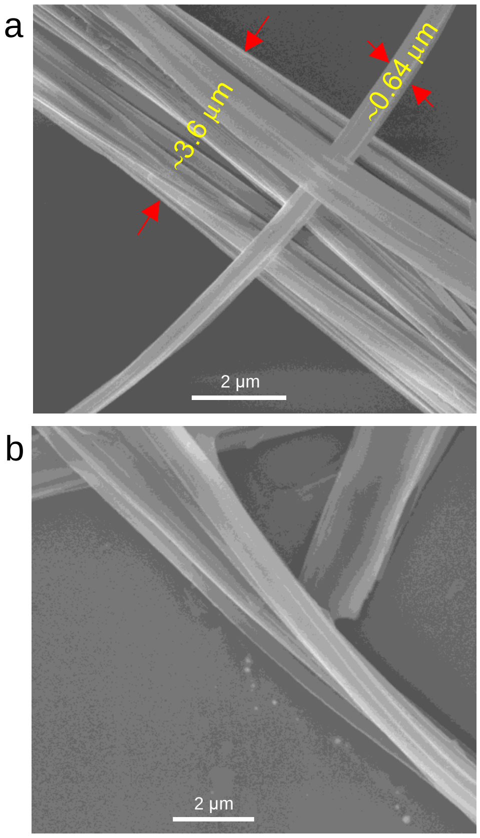

Figure S28. SEM images for the washed samples of self-assembled Ag_9-NCs (2.0 wt%) in 50 wt% $C_{12}E_4$ at the presence of 1.5 wt% ^{L}Val (a) and ^{D}Val (b), which highlight the secondary self-assembly of the ribbons.

References

[1] Z. Xie, P. Sun, Z. Wang, H. Li, L. Yu, D. Sun, M. Chen, Y. Bi, X. Xin, J. Hao, *Angew. Chem. Int. Ed.* **2020**, *59*, 9922-9927.

[2] Y. Bi, Z. Wang, T. Liu, D. Sun, N. Godbert, H. Li, J. Hao, X. Xin, *ACS Nano* **2021**, *15*, 15910-15919.

[3] N. Feng, Z. Wang, D. Sun, P. Sun, X. Xin, X. Cheng, H. Li, *Adv. Optical Mater.* **2022**, *10*, 2102319.

[4] J. Shen, J. Pang, T. Kalwarczyk, R. Hołyst, X. Xin, G. Xu, X. Luan, Y. Yang, *J. Mater. Chem. C* **2015**, *3*, 8104-8113.

[5] S. Tang, T. Yang, Z. Zhao, T. Zhu, Q. Zhang, W. Hou, W. Z. Yuan, *Chem. Soc. Rev.* **2021**, *50*, 12616-12655.
